# Proteome profiling of whole plasma and plasma-derived extracellular vesicles facilitates the detection of tissue biomarkers in the non-obese diabetic mouse

**DOI:** 10.3389/fendo.2022.971313

**Published:** 2022-09-28

**Authors:** Isabel M. Diaz Lozano, Helena Sork, Virginia M. Stone, Maria Eldh, Xiaofang Cao, Maria Pernemalm, Susanne Gabrielsson, Malin Flodström-Tullberg

**Affiliations:** ^1^ Center for Infectious Medicine, Department of Medicine Huddinge, Karolinska Institutet, Karolinska University Hospital Huddinge, Stockholm, Sweden; ^2^ Institute of Technology, University of Tartu, Tartu, Estonia; ^3^ Department of Clinical Immunology and Transfusion Medicine and Division of Immunology and Allergy, Department of Medicine Solna, Karolinska University Hospital and Karolinska Institutet, Stockholm, Sweden; ^4^ Department of Oncology and Pathology/Science for Life Laboratory, Karolinska Institutet, Stockholm, Sweden

**Keywords:** biomarkers, extracellular vesicles, type 1 diabetes, mass spectrometry, plasma, exosome, mouse, non-diabetic (NOD) mouse

## Abstract

The mechanism by which pancreatic beta cells are destroyed in type 1 diabetes (T1D) remains to be fully understood. Recent observations indicate that the disease may arise because of different pathobiological mechanisms (endotypes). The discovery of one or several protein biomarkers measurable in readily available liquid biopsies (e.g. blood plasma) during the pre-diabetic period may enable personalized disease interventions. Recent studies have shown that extracellular vesicles (EVs) are a source of tissue proteins in liquid biopsies. Using plasma samples collected from pre-diabetic non-obese diabetic (NOD) mice (an experimental model of T1D) we addressed if combined analysis of whole plasma samples and plasma-derived EV fractions increases the number of unique proteins identified by mass spectrometry (MS) compared to the analysis of whole plasma samples alone. LC-MS/MS analysis of plasma samples depleted of abundant proteins and subjected to peptide fractionation identified more than 2300 proteins, while the analysis of EV-enriched plasma samples identified more than 600 proteins. Of the proteins detected in EV-enriched samples, more than a third were not identified in whole plasma samples and many were classified as either tissue-enriched or of tissue-specific origin. In conclusion, parallel profiling of EV-enriched plasma fractions and whole plasma samples increases the overall proteome depth and facilitates the discovery of tissue-enriched proteins in plasma. If applied to plasma samples collected longitudinally from the NOD mouse or from models with other pathobiological mechanisms, the integrated proteome profiling scheme described herein may be useful for the discovery of new and potentially endotype specific biomarkers in T1D.

## 1 Introduction

Type 1 diabetes (T1D) is a common, life-threatening, chronic disease which arises due to the loss of insulin-producing pancreatic beta cells. The disease is initiated in an asymptomatic period when autoantibodies to beta cell antigens can be detected in the blood. This pre-diabetic period can last from a few months to several years after which T cells are thought to infiltrate the pancreatic islets and attack and destroy beta cells. This leads to insufficient insulin production and the development of severe hyperglycemia (1). Recent observations indicate that T1D is heterogenous and can occur through several different disease mechanisms, so-called endotypes (2). The discovery of one or several disease specific markers in the pre-symptomatic stage of T1D could enable endotype-driven preventive treatments.

Biomarkers are measurable factors that indicate the presence, progression, or severity of disease. In recent years, the interest in identifying biomarkers present in liquid biopsies (such as plasma and urine) has increased substantially, especially in diseases affecting internal organs and in cases where non-invasive techniques for disease monitoring are highly desirable (e.g., profiling of solid tumors (3), staging of T1D, etc). C-peptide is a molecule which is cleaved from the pro-insulin molecule during insulin production and is secreted along with insulin. This protein is commonly used to estimate endogenous insulin production in individuals undergoing insulin therapy. The fact that C-peptide can be measured in blood and urine shows that proteins released from beta cells can be detected in easily accessible body fluids. It is possible that other proteins released by beta cells or the pancreas, either in their native form or when post-translationally modified, could mark a pathological stage in the development of T1D.

Non-obese diabetic (NOD) mice spontaneously develop T1D and share many similarities with the human disease, such as polymorphisms in major histocompatibility complex (MHC) class II and the development of both B- and T-cell islet autoimmunity. Hyperglycemia develops after the animals have reached 10-12 weeks of age and is a result of CD8^+^ T cell mediated destruction of beta cells. As in humans, the appearance of islet autoantibodies (e.g. GADA, IA-2A, and IAA) in the bloodstream of NOD mice marks the onset of the pre-diabetic phase (Stage 1) and their presence strongly predicts the likelihood of clinical disease onset (4). Diabetes incidence can be altered in NOD mice after exposure to environmental factors suspected to modulate the risk of T1D in humans. One example is infection by Coxsackie B viruses, a group of viruses linked to the development of diabetes in humans (5), and which accelerate diabetes development in NOD mice (6–8). Moreover, it is also possible to genetically change NOD mice to assess, for example, the effect of certain polymorphisms on the autoimmune process [e.g (4, 9, 10)]. As such, and combined with our increased knowledge surrounding different endotypes in human T1D, this model may be of use for the discovery of new disease predictive biomarkers.

Proteomic analysis of blood plasma using mass spectrometry (MS)-based methods is increasingly used in the discovery of biomarkers and allows us to further extend our knowledge of disease mechanisms (11). The blood plasma proteome consists of proteins involved in basic plasma functions including, but not limited to, the transport of nutrients and hormones to various parts of the body, maintenance of blood pressure and blood volume, coagulation and immunity. Plasma also contains waste products and sometimes proteins are found that reflect disease states associated with tissue dysfunction or malignancy. The latter category of proteins may be low in abundance and their detection by MS often requires the prior removal of the most abundant plasma proteins followed by fractionation or the use of other targeted approaches (11).

Enrichment of the extracellular vesicle (EV) subfraction of plasma and subsequent EV proteome analysis may also allow for improved detection and identification of low abundance biomarkers (11). EVs are small phospholipid bilayer membrane-bound vesicles that are secreted by all cell types into the extracellular space. They contain cargo such as DNA, RNA and proteins derived from their parent cell and may have important functions in intracellular communication (12). If released from transformed, infected or functionally damaged cells, their contents might also reflect changes occurring at the level of a disease-affected organ. As such, the analysis of EVs in body fluids could provide a method to enrich and facilitate the discovery of tissue specific disease biomarkers, including in T1D.

EV subtypes are distinguished based on their size or the pathways through which they are produced and released; exosomes (~30-150 nm) are generated through the endocytic pathway; microvesicles or ectosomes (~100–1000 nm) are produced by outward budding of the plasma membrane; and apoptotic bodies (50-5000 nm) are formed during apoptotic membrane blebbing (12, 13). Although no specific protein marker(s) have been found that can be used to distinguish or selectively isolate a specific type of vesicles, exosomes often carry proteins that reflect their endosomal origin, including members of the ESCRT family, accessory proteins (e.g., Alix and TSG101) and tetraspanins (e.g. CD9, CD63 and CD81) (12, 13). When it comes to biomarker studies apoptotic bodies are commonly excluded and from here onwards, the term EVs will only refer to exosomes and microvesicles.

Several methods have been developed for the isolation or enrichment of EVs and the choice of method often depends on the type and volume of the starting material (14). Differential ultracentrifugation (UC) has been widely used to separate exosomes and microvesicles from both cell debris and apoptotic bodies in large volumes of study material (such as cell culture media or body fluids like urine) (12, 15). This method however has some limitations including the co-purification of protein aggregates, long processing time and the need for expensive equipment (16). Other methods are increasing in popularity due to benefits including their less time-consuming nature, the fact they can be used with smaller sample volumes, are more specific for the isolation of small and large EVs and they are associated with reduced sample loss and EV aggregation. These methods include size exclusion chromatography (SEC), membrane affinity (MA) and precipitation methods including immunoprecipitation [e.g. in (14)].

In this study, we investigated whether simultaneous MS-based analysis of whole plasma samples and EV proteomes from NOD mouse plasma is complementary and if it increases the number of unique proteins detected. Initially, we performed a systematic comparison of two methods for the enrichment of EVs from plasma samples collected from pre-diabetic NOD mice. We also introduced additional pre-analytical steps to improve the overall proteome depth of LC-MS/MS-based detection of unique proteins in both whole plasma and EV-enriched plasma samples. Our studies suggest that the inclusion of EV proteome analysis increases the detection of tissue- and cell specific proteins in NOD mouse plasma samples.

## 2 Materials and methods

### 2.1 Animal husbandry

Non-obese diabetic (NOD) mice were bred and housed in a specific pathogen-free environment at the PKL animal facility, Karolinska University Hospital Huddinge, Sweden. Experiments were approved by the local ethics committee (Linköpings Försöksdjursetiska Nämnd, Linköping, Sweden) and conducted in accordance with the NIH principles of laboratory animal care and national laws in Sweden. Mice were not single housed and a maximum of 5 animals were kept in the same ventilated cage. Food and water were provided *ad libitum*.

### 2.2 Blood glucose level measurements

Blood glucose concentrations in non-fasted animals were measured in blood from the tail vein using a Bayer Contour XT blood glucose meter (Bayer, Switzerland). Diabetes was defined as a blood glucose value > 18 mmol/L or two measurements made on consecutive days between 13 – 18 mmol/L.

### 2.3 Plasma collection

Blood samples were collected by terminal heart puncture (through the chest) under deep inhalation anesthesia (isoflurane) using a 23G needle and a 1 mL syringe coated with 12 mM EDTA solution (in PBS). Blood samples were transferred to pre-coated BD Microtainer K2 EDTA tubes (Becton Dickinson AB, USA) and plasma was obtained by two consecutive centrifugations. First, blood was spun at 2,000 x g for 10 min at 4°C. The supernatant was then transferred to a new Eppendorf tube and spun at 3,000 x g for 30 min at 4°C to remove platelets and cell debris. Only plasma samples without hemolysis were used. A plasma sample was deemed to be hemolyzed according to visual inspection of the plasma color using a threshold of 20 mg dL^-1^ hemoglobin plasma (17). Plasma samples were used either directly after collection (fresh) or they were aliquoted and stored at -80°C before further processing and/or analyses.

### 2.4 Plasma EV enrichment by membrane affinity-based method (exoEasy)

EVs were enriched from either fresh or frozen mouse plasma using the exoEasy Maxi kit (QIAGEN, USA) according to the manufacturer’s protocol. Briefly, plasma was filtered using a 0.8 μM syringe filter (Sartorius AG, Germany) to remove large particles and mixed with an equivalent volume of XBP buffer. The plasma/XBP mix was added to an exoEasy spin column and spun at 500 x g for 1 min at room temperature (RT). Then the column was washed with 10 mL of XWP buffer and spun at 3,000 x g for 1 min at RT to remove residual buffer. The EVs were eluted by adding 400 μL of XE buffer to the membrane and after a 1-minute incubation the column was spun at 500 x g for 5 minutes at RT to collect the elute. The elute was re-applied to the exoEasy spin column membrane followed by a 1-minute incubation at RT. Finally, the column was spun at 5,000 x g for 5 min at RT to collect the final elute.

### 2.5 Plasma EV enrichment by size exclusion chromatography (qEV)

EVs were enriched either from fresh or frozen mouse plasma pooled from 2-3 animals using Izon qEVoriginal/70nm Size Exclusion Columns (Izon Science Ltd., New Zealand). Prior to column loading, the plasma was filtered through a 0.8 μm syringe filter (Sartorius AG, Germany) to remove large particles. Thereafter the plasma (0.5 – 0.8 mL) was loaded onto a qEVoriginal column for size exclusion-based EV enrichment. Fractions of 0.5 mL were collected and analyzed for protein (see *Protein quantification*) and particle (see *Nanoparticle tracking analysis*) content to identify the optimal fractions for EV collection. For size, morphology and proteome analyses, fractions 7-10 were pooled and kept at -80°C until further processing.

### 2.6 Protein quantification

Protein concentrations were analyzed with the Pierce BCA Protein Assay Kit (Thermo Fisher Scientific, USA). Optical densities were measured by a Bio-Rad xMark Microplate Spectrophotometer (Bio-Rad Laboratories, USA) at 562 nm. A standard curve was generated using a second order polynomial (quadratic) fit in GraphPad PRISM 8 (version 8.1.2, San Diego, USA). The protein content of each sample (exoEasy) or fraction (qEV, individual or pooled) was determined by extrapolating the measured optical densities to the obtained standard curve.

### 2.7 Transmission electron microscopy

Transmission electron microscopy *(*TEM) was performed using EVs enriched from plasma samples. For EVs enriched using exoEasy, 20 μL of the elute was fixed with 5 μL of 4% formaldehyde solution. For EVs enriched using qEV, each 7-10 fraction pool was first concentrated to a final volume of 100-120 μl using 10kDa Amicon Ultra-0.5 mL Centrifugal Filter Units (Millipore, USA). After the concentration step, a 20 μL aliquot was fixed with 5 μL of 4% formaldehyde solution. Five μL EV aliquots from samples isolated by both methods were applied onto a Formvar/Carbon type B coated electron microscopy grid (Ted Pella Inc., USA) which was then washed with double distilled H_2_O, blotted dry with filter paper and stained with 2% uranyl acetate (Thermo Fisher Scientific, USA). The grid was again blotted dry and analyzed with a FEI Tecnai 10 transmission electron microscope (FEI, USA) at an accelerating voltage of 100 kV.

### 2.8 Nanoparticle tracking analysis

Particle concentration and size distribution of the plasma-derived EV preparations were measured by nanoparticle tracking analysis (NTA) using the Nano Sight NS500 nanoparticle analyzer (Malvern Instruments, UK). The plasma-derived EV samples isolated either by qEV or exoEasy were diluted in PBS (1:100) and mixed before sample injection in the chamber at a fixed flow rate. A total of five videos of 30 seconds were recorded and then analyzed using the NTA Software 2.3 (Malvern Instruments, UK) at detection threshold 7, camera gain 400, camera level 7, shutter length 19.9688 ms and shutter setting 1.0 for optimal visualization of the maximum number of EVs. The NTA post-acquisition settings were kept constant between the samples.

### 2.9 Depletion of abundant plasma proteins

For studies addressing whether the removal of abundant plasma proteins would increase the number of proteins detected by LC-MS/MS, aliquots of plasma and plasma-derived EV samples were subjected to the removal of three highly-abundant plasma proteins using the Multiple Affinity Removal Spin Cartridge Mouse-3 (Agilent Technologies, USA) before liquid chromatography with tandem mass spectrometry (LC-MS/MS) analysis. In brief, 25 μL aliquots of whole plasma were diluted in 175 μL of depletion Buffer A (Agilent Technologies, USA) and filtered through a 0.22 μM cellulose acetate spin filter (Agilent Technologies, USA) by centrifugation at 15,000 x g for 2 min at RT.

EV samples (2 mL of fraction 7 to 10 from qEV and 0.4 mL exoEasy elute) were sonicated in an ultrasonic bath in 3 cycles of 5 min and split into two aliquots of equal volume. Each aliquot was precipitated with ice-cold 10% Trichloroacetic acid (TCA) solution in acetone (1:1 v/v) overnight at -20°C. The mixture was then spun at 15,000 x g for 10 minutes at 4°C. The supernatant was discarded, and the pellet was washed three times in pure ice-cold acetone. After washing, the pellet was dried under the fume hood. Next, one aliquot from each sample (qEV and exoEasy) was re-suspended in 100 μL of depletion Buffer A. The other aliquot was re-suspended in 100 μL of lysis buffer containing 1% β-octyl-glucoside, 6M urea and PBS.

Samples diluted in depletion Buffer A (190 μL of diluted and filtered plasma and 100 μL of either qEV or exoEasy samples) were loaded onto the Multiple Affinity Removal System (MARS Mouse-3) cartridge (Agilent Technologies, USA) and the flow-through fraction was collected by centrifugation for 2 min at 100 x g at RT. Two washes, using 400 μL of Buffer A were carried out to obtain maximum sample yield. The flow-through and wash fractions were combined. The spin cartridge was washed with 2 mL of washing Buffer B (Agilent Technologies, USA) to remove bound proteins and was then re-equilibrated with Buffer A. The combined fractions were dried using a speed vac and resuspended in 100 μL of lysis buffer containing 1% β-octyl-glucoside, 6M urea and PBS. Thereafter, the protein concentrations of each depleted and non-depleted whole plasma sample were estimated using the DC Protein Assay Kit, Bio-Rad Laboratories, USA) using bovine serum albumin (BSA) for the standard curve. For qEV and exoEasy EVs samples (depleted and non-depleted), the protein concentration was measured using the Dot-it-Spot-it^®^ protein assay (Maplestone AB, Sweden) with a detection range of 0.15 – 5 ng BSA per dot. Samples were then subjected to LC-MS/MS analysis.

For HiRIEF LC-MS/MS analyses of whole plasma, samples were first depleted of highly abundant plasma proteins using a Multiple Affinity Removal Spin Cartridge Mouse-3 (Agilent Technologies, USA). The previously described method was used along with further modifications. Briefly, samples (aliquots of 30 μL) were diluted in 170 μL of Buffer A supplemented with protease inhibitors (cOmplete^™^, Mini, EDTA-free Protease Inhibitor Cocktail, Roche, Switzerland) and filtered through a 0.22 μM cellulose acetate spin filter. The column was washed with 400 μL of Buffer A prior to sample application (190 μL of diluted and filtered plasma). The flow-through fraction F1 was collected after centrifugation at 50 x g for 2 min at RT (to avoid drying out of the resin bed). The column was then washed twice with 400 μL of Buffer A and centrifuged at 100 x g for 2 min at RT. The flow-through fractions were collected in the same tube as the F1 fraction. The spin cartridge was washed with 2 mL of Buffer A, to eliminate bound proteins, and was re-equilibrated with 2 mL of Buffer A. The depleted fractions were subjected to a buffer exchange by using a 5K MWCO spin concentrator (Agilent Technologies, USA; centrifuged at 750 x g for 20 min at RT a total of 3 times) followed by dilution in 4 mL of HEPES (50 mM, pH 7.6) buffer. The total protein amount was estimated using the DC Protein assay kit (Bio-Rad Laboratories, USA).

### 2.10 Protein digestions, liquid chromatography-mass spectrometry, data processing and analysis

Samples were digested and analyzed by LC-MS/MS according to three different protocols. The first method was used for whole plasma and EV samples (enriched using either exoEasy or qEV) subjected to or excluded from the depletion of abundant plasma proteins according to section *Depletion of abundant plasma proteins*. In brief, non-depleted control samples (whole plasma and EVs, 20 μg aliquots of each) and depleted samples underwent reduction and alkalinization prior to on-filter digestion with trypsin-Lys-C mixture then purification using Pierce C18 Spin Columns (Thermo Fisher Scientific, USA) and drying using a speedvac system. Finally, samples were dissolved in either 50 μL or 21 μL (qEV samples) of 0.1% formic acid. The samples were then analyzed by label free LC-MS/MS quantification. Peptides were separated in reverse-phase on a C18-column with a 90 min long gradient, and electro sprayed on-line to a QEx-Orbitrap mass spectrometer (Thermo Fisher Scientific, USA). Tandem mass spectrometry was performed applying HCD fragmentation. Data was analysed using the Sequest algorithm, embedded in Proteome Discoverer 1.4 (Thermo Fisher Scientific, USA) with specificity for proteins from the *Mus musculus* proteome extracted from Uniprot (2018–05). Database searches for peptides extracted from EV-enriched samples using the first digestion method were performed using the software MaxQuant 1.5.1.2 and a search against the *Mus musculus* proteome was extracted from Uniprot, release January 2019. The search parameters were set to Taxonomy: *Mus musculus*, Enzyme: Trypsin. Fixed modification was set to carbamidomethyl (C), and the selections for variable modifications were oxidation (M) and deamidated (NQ). The search criteria for protein identification were set to at least two matching peptides of 95% confidence level per protein. Digestions and the following mass spectrometry analysis were performed by the mass spectrometry facility at Uppsala University, Uppsala, Sweden.

The second method was applied to EVs isolated by qEV from an 800 μL pool of plasma made from two NOD mice. The digestions and following mass spectrometry analysis were performed by the Clinical Proteomics Mass Spectrometry facility, Karolinska Institutet/Karolinska University Hospital/Science for Life Laboratory, Stockholm, Sweden. Samples were dissolved in PBS and prepared for mass spectrometry analysis using a modified version of the SP3 protein clean up and digestion protocol (18). In brief, 20 μg of protein was alkylated with 4 mM chloroacetamide and mixed with 22 μL of Sera-Mega SP3 bead with the addition of 100% acetonitrile (final concentration 60%). The mix was rotated for 30 min at RT, then placed on a magnetic rack and the supernatant was discarded. It was then washed twice with 70% ethanol and once with 100% acetonitrile. The bead-protein mixture was reconstituted in 100 μL trypsin buffer (50 mM HEPES pH: 7.6 and 1:50 trypsin to protein ratio (weight to weight)) and incubated overnight at 37°C, which was followed by SP3 peptide clean up. An aliquot of approximately 25 μg of digested peptides was suspended in LC mobile phase A and ~4 μg analyzed by label free LC-MS/MS quantification. Online LC-MS was performed as previously described (19) using a Thermo Fisher Scientific Dionex nano LC-system in a 3 hr 5-40% ACN gradient coupled to Thermo Fisher Scientific High Field QExactive-HF. The obtained MS raw files were analyzed using Sequest-Percolator or Target Decoy PSM Validator under the software platform Proteome Discoverer 1.4 (Thermo Fisher Scientific, USA) against the *mouse* Uniprot database and filtered to a 1% FDR cut off. The algorithm considered tryptic peptides with maximum 2 missed cleavages. Carbamidomethylation (C) was set as fixed modifications, and oxidation (M) and deamidation (N) as variable modifications. The MS-data have been deposited to the ProteomeXchange Consortium *via* the PRIDE (20) partner repository with the dataset identifier PXD033867.

The third method was used for samples depleted of highly abundant plasma proteins which were subsequently analyzed by HiRIEF LC-MS/MS. Digestions and the following mass spectrometry analysis were performed by the Clinical Proteomics Mass Spectrometry Facility, Karolinska Institutet/Karolinska University Hospital/Science for Life Laboratory, Stockholm, Sweden. The proteins in the samples were digested using an in-solution digestion protocol with LysC (Lysyl Endopeptidase, FUJIFILM Wako Chemicals, USA) and trypsin (sequencing grade modified, Thermo Fisher Scientific, USA). In brief, 200 μg protein from each sample was alkylated with 8 mM chloroacetamide. The protein mixture was reconstituted in 100 μL of LysC buffer (0.5 M Urea, 50 mM HEPES pH: 7.6 and 1:50 LysC to protein ratio) and incubated overnight at 37°C. Trypsin was then added at a 1:50 enzyme to protein ratio in 100 μL of 50 mM HEPES pH 7.6 and incubated overnight at 37°C. The samples were then pH adjusted using TEAB buffer pH 8.5 (100 mM final concentration). Finally, 100 μg of peptides from each sample were labelled with isobaric TMT-tags (TMT10plex reagent) according to the manufacturer’s protocol (Thermo Fisher Scientific, USA) and separated by an immobilized pH gradient - isoelectric focusing (IPG-IEF) on 3-10 strips as described previously (19). Extracted peptide fractions from the IPG-IEF were separated using an online 3000 RSLCnano system coupled to a Thermo Fisher Scientific Q Exactive-HF. MSGF+ and Percolator in the Galaxy platform were used to match MS spectra to the *Mus musculus* protein database (UniProt, filtered and reviewed) (21). The MS-data have been deposited to the ProteomeXchange Consortium *via* the PRIDE (20) partner repository with the dataset identifier PXD033867.

### 2.11 Data analysis and bioinformatics

#### 2.11.1 EV protein database searches

The presence of EV protein markers in the MS data set was confirmed by matching the complete data with the list of top 100 proteins identified in EVs according to Vesiclepedia (Version 4.1, 2018) (http://microvesicles.org/).

#### 2.11.2 Gene ontology, principal component analysis and functional enrichment analysis

Gene ontology (GO) of the identified proteins from plasma and plasma EVs enriched by SEC was carried out using the Database for Annotation, Visualization and Integrated Discovery (DAVID) v6.8 (https://david.ncifcrf.gov/) (22). The gene list (gene symbol) for *Mus musculus* was submitted with the application of a threshold of 2 counts, EASE score threshold of 0.1, Benjamini and Hochberg FDR of <0.05 and maximum number of Ids in each category of 1000. GO enrichment analysis for biological process, cellular component and molecular function terms was conducted. PCA analysis for batch or sampling effect on HiRIEF LC/MS-MS plasma proteome was performed using R (version 4.0.4). The search for specific-tissue proteins was conducted in DAVID v6.8 using the Functional Enrichment Analysis tool and selection “Tissue_Expression/Up_Tissue” option for both whole plasma and plasma-derived EV protein lists. The functional annotation chart was then used by applying the same set up enlisted for GO analysis as described above. The top 10 most enriched tissues were ranked according to their p-values. It should be noted that from a total of 2342 proteins discovered in whole plasma samples, DAVID recognized 2284 gene entries. The corresponding numbers for proteins detected in plasma derived EVs were 640 and 602, respectively. By reviewing the missing entries manually, it was observed that many proteins not recognized in the EV enriched samples correspond to immunoglobulins (**Table S6**). Tissue specific gene and/or protein expression of selected proteins was explored using the translational human pancreatic islet genotype tissue-expression resource (TIGER) data portal (http://tiger.bsc.es) (23), and the human protein atlas (HPA, www.proteinatlas.org) (24–26) data base.

#### 2.11.3 Bioinformatic analysis

**Figure 1:**
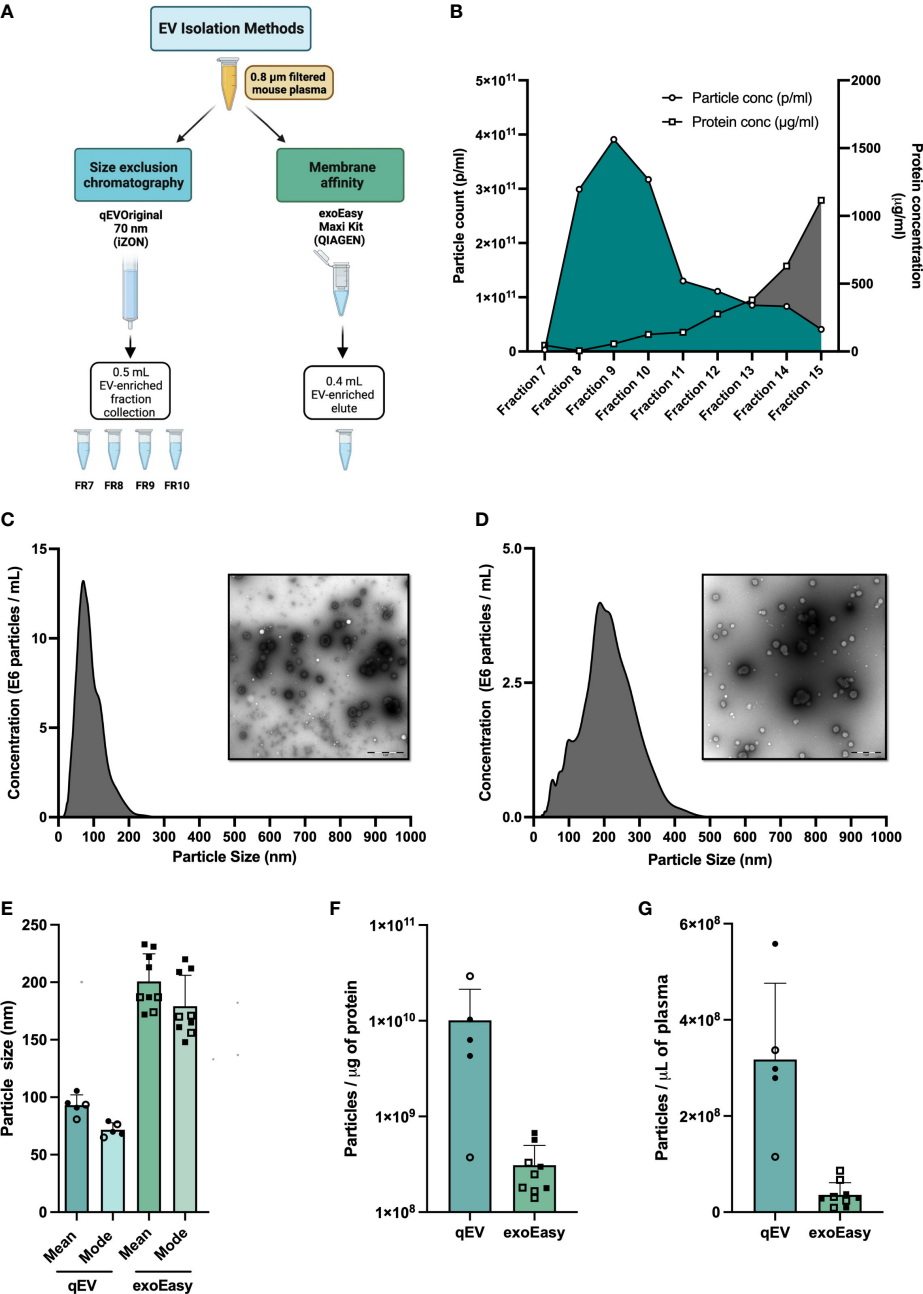
Extracellular vesicle (EV) enrichment and characterization. **(A)** Plasma collection workflow. Blood samples from pre-diabetic NOD mice were collected in EDTA tubes. Plasma was isolated and filtered through a 0.8 μM filter prior to EV enrichment. EVs were enriched using one of two isolation methods, Size Exclusion Chromatography (SEC) using the qEVoriginal 70 nm column (iZon), or Membrane affinity (MA) using exoEasy Maxi spin columns (Qiagen). **(B)** Fresh plasma samples (560 or 900 μL) were applied to qEV original 70 nm columns. Fractions 7 to 15 were collected and particle count and protein content in each fraction were analysed by nanoparticle tracking analysis (NTA) and BCA assay, respectively. Shown is a representative graph made from the analysis of 900 μL of plasma. **(C, D)** Size distribution (nm) of particles (690 μL fresh plasma for each experiment) enriched by qEVoriginal 70 nm column, pooled fractions 7-10 **(C)** or exoEasy spin column **(D)** with representative TEM pictures. Scale bar size 1 μM. **(E–G)** Plasma was collected from non-diabetic NOD mice and was enriched for EVs using qEV (pooled fractions 7-10) or exoEasy. **(E)** Average mean and modal size of the enriched particles as determined by NTA (NanoSight NS500). **(F)** Average particle number per microliter of mouse plasma used to isolate EVs by qEV or exoEasy. **(G)** Ratio of the average of particle number (measured by NanoSight NS500) and protein amount (Pierce BCA Protein Assay kit, Thermo Fisher Scientific, USA) for qEV or exoEasy enriched EVs. Bars represent mean and standard deviation of a total of 9 and 5 independent enrichments using qEV and exoEasy, respectively. (Open circles and squares correspond to EV samples enriched using frozen plasma samples (qEV, n = 2, exoEasy, n = 5); closed circles and squares correspond to EV samples isolated from fresh plasma samples (qEV, n = 3; exoEasy, n = 4).

Microsoft Excel was used to make radar charts in **Figures 3B** and **4C–E**. R (version 4.0.4) and GraphPad Prism v.9 were used to plot graphs and BioRender (BioRender.com) .for methods description (**Figure 1A**).

## 3 Results

### 3.1 Blood collection and plasma extraction

The procedure for sample collection and processing may affect sample quality and compromise the detection of disease biomarkers with low abundance (27). For example, lysis of red blood cells (hemolysis) may occur during the collection and handling of blood samples (28). Moreover, the abundance of EVs is typically higher in serum than in plasma due to the platelet-derived EVs that are released during clot formation when preparing the serum (29). Through the use of anticoagulants, the release of platelet-derived EVs can be largely avoided. A recent study suggested that EDTA is superior to acid citrate dextrose or citrate, in preventing coagulation and contamination of platelet-derived EVs (30). As a result of these findings, blood was harvested from pre-diabetic female NOD mice *via* heart puncture (trough the chest) using a syringe pre-coated with EDTA. Plasma was extracted as described in *Materials and Methods*. Care was taken to avoid hemolysis and if a plasma sample was hemolyzed it was not used in the study. Using this method, we were able to obtain up to 1200 μL of whole blood per animal, which corresponds to 650 μL of plasma.

### 3.2 Comparisons between membrane affinity- and size exclusion chromatography-based enrichment of murine plasma EVs

The method of choice for EV enrichment is largely determined by the amount of biological sample available. For studies using mice or samples from small children, the plasma volume is limited, especially if individual samples rather than pooled samples are analyzed. In initial studies (not shown), we explored the feasibility of using UC for EV enrichment. Due to a poor EV yield and high amounts of co-enriched plasma proteins (data not shown), we did not proceed with this method. Instead, we compared two other methods that were more suited for use in enriching EVs from the small plasma volumes that can be obtained from mice (**Figure 1A**). The first, exoEasy, is based on a membrane-based affinity (MA) binding step, and the second, qEV, on size exclusion chromatography (SEC) (**Figure 1A**). Particles were isolated according to the manufacturer’s protocols and initially analyzed for particle yield, size and protein content. For qEV we began by testing two different volumes of plasma (560 and 900μl). We collected the elute fractions 7-15 and analyzed particle numbers and protein yield in each to determine which fractions had the highest number of particles and the least amount of protein. The relative distribution of particles and protein content in each fraction was similar between the two samples (**Figure 1B** and data not shown). Due to their high particle and low protein content, fractions 7-10 were the fractions of interest and for all further analyses these fractions were collected and pooled.

After NTA analysis of the EV-enriched samples we found that the exoEasy method enriched for larger but fewer particles than qEV, regardless of whether fresh or frozen plasma was used (**Figures 1C–F**). We also noted when comparing the number of particles per microgram of protein, qEV gave higher values than exoEasy (**Figure 1G**). Electron microscopy (EM) images mirrored the results from the NTA analysis and supported the observation that exoEasy enriches particles with larger size than qEV (**Figures 1C, D**, **Figure S1**). Moreover, EVs with cup-shaped morphology were seen in the EM images but it was not possible to clearly distinguish the EVs from lipoproteins, which are commonly co-isolated with EVs from plasma samples (31). Of note, the exoEasy-enriched samples had a greater fraction of larger particles without a defined, spherical shape than the qEV-enriched samples.

### 3.3 Depletion of highly abundant plasma proteins from exoEasy and qEV enriched murine plasma EVs does not increase protein coverage by LC-MS/MS based proteomics analysis

**Figure 2:**
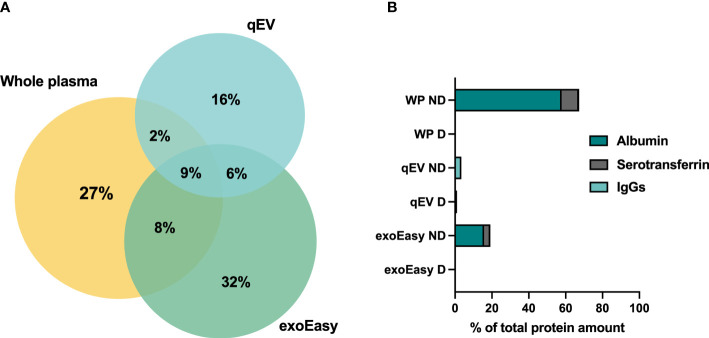
Protein overlaps between whole plasma samples and EV-enriched plasma fractions analyzed by LC-MS/MS, and the presence of abundant plasma proteins before and after depletion. **(A)** Plasma was collected from pre-diabetic female NOD mice. LC-MS/MS analysis was performed on 20 μL of frozen plasma sample or on EVs enriched from 500 μL of frozen plasma by qEV or exoEasy. Venn diagram representing the percentages of shared and unique proteins identified by MS in whole plasma sample (number of proteins = 298), and EV samples enriched by qEV (n = 215) or exoEasy (number of proteins = 356). **(B)** Plasma was collected from non-diabetic NOD mice, frozen and pooled before analysis. A whole plasma (WP) sample or EV samples enriched using qEV (n = 2, pooled or separate) or exoEasy (n = 2) were either untreated or subjected to removal of three high-abundance/highly-abundant plasma proteins using the Multiple Affinity Removal Spin cartridge mouse-3 column (Agilent Technologies, USA). LC-MS/MS analysis was performed and the percentages of the total protein amount that accounted for albumin, serotransferrin and IgGs in whole plasma (WP), qEV and exoEasy EV samples with **(D)** and without (ND) depletion is shown (n =1 out of 1-2 for each comparison).

We next performed LC-MS/MS analysis on EVs enriched by exoEasy or by qEV and for further comparison also included a whole plasma sample. MS analysis revealed a partial overlap in proteins detected between the EV samples and the overlap was greater between whole plasma and exoEasy samples than whole plasma and qEV samples (**Figure 2A**). The top 10 most abundant proteins detected in the EV samples were proteins commonly found in plasma such as serum albumin and the lipoproteins ApoB and ApoA which are typically detected in plasma derived EV samples (31, 32) (**Table 1**, left column). It is worth noting that the amount of albumin was particularly high in EVs enriched using exoEasy.

**Table 1:** Top 10 most abundant proteins detected by LC-MS/MS in whole plasma or EV enriched samples from mouse plasma by exoEasy or qEV, without (non-depleted) or after (depleted) the removal of abundant plasma proteins using the Multiple Affinity Removal Spin Cartridge Mouse-3 column.

	exoEasy (non-depleted)	exoEasy (depleted)
1	Serum albumin	Prothrombin
2	Prothrombin	Prothrombin
3	Inter-alpha trypsin inhibitor chain 1	Cfd protein
4	Inter-alpha trypsin inhibitor chain H2	Coagulation factor X
5	Inter-alpha trypsin inhibitor chain H3	Vitronectin
6	Thrombospondin-1	Complement factor H
7	Serotransferrin	Apolipoprotein A-IV
8	Pregnancy zone protein	Kininogen-1
9	Murinoglobulin-1	Proz protein
10	Serine protease inhibitor A3K	Coagulation factor V
	**qEV (non-depleted)**	**qEV (depleted)**
1	Fibrinogen beta chain	Apolipoprotein-A
2	Fibrinogen gamma chain	Fibrinogen beta chain
3	Apolipoprotein B-100	Complement factor H
4	Fibrinogen alpha chain	Coagulation factor XIII B
5	Pregnancy zone protein	Fibrinogen alpha chain
6	Fibronectin	Fibrinogen gamma chain
7	Apolipoprotein E	C4b-binding protein
8	Immunoglobulin heavy constant mu^1^	Serum albumin
9	Apolipoprotein A-IV	Serglycin
10	Murinoglobulin-1	Apolipoprotein E
	**Whole plasma (non-depleted)**	**Whole plasma (depleted)**
1	Serum albumin	Pregnancy zone protein
2	Serotransferrin	Murinoglobulin-1
3	Pregnancy zone protein	Alpha-1 antitrypsin 1-3
4	Murinoglobulin-1	Serine protease inhibitor A3K
5	Complement C3	Serpina1a protein
6	Fibrinogen gamma chain	Alpha-1-antitrypsin 1-4
7	Apolipoprotein A-I	Serpina1 DOM-7
8	Fibronectin	Complement C3
9	Serine protease inhibitor A3K	Fibrinogen beta chain
10	Fibrinogen beta chain	Serine protease inhibitor A3M

A common procedure employed to improve LC-MS/MS analysis of whole serum or plasma samples is the removal of highly-abundant plasma proteins using, for example, an affinity column. This improves the detection of proteins with a low abundancy (33–35). It is possible to decrease the abundance of three common plasma proteins (albumin, transferrin and IgG) from mouse plasma samples using a commercially available affinity removal column. To assess whether this type of column could be used to lower the relative abundance of the aforementioned proteins and increase the overall protein coverage by LC-MS/MS analysis, the EV samples were analyzed with and without prior depletion. A whole plasma sample was included as a positive control. Depletion efficiently decreased the presence of the most abundant plasma proteins in all samples (**Figure 2B**, **Table 1**). Moreover, the removal of these proteins increased the protein coverage in the plasma but failed to do so in the EV samples (**Figure S2**). Prior removal of abundant plasma proteins from EV fractions was therefore not further pursued.

Up to this point, our analyses showed that EV enrichment from mouse plasma samples using qEV and exoEasy resulted in the isolation of particles that were somewhat different from one another. As our goal was to have as little overlap as possible with the whole plasma proteome, the qEV isolation method was deemed a better choice for our research efforts and was therefore used for the rest of the study.

### 3.4 Global proteome analysis of plasma EVs from pre-diabetic NOD mice

**Figure 3:**
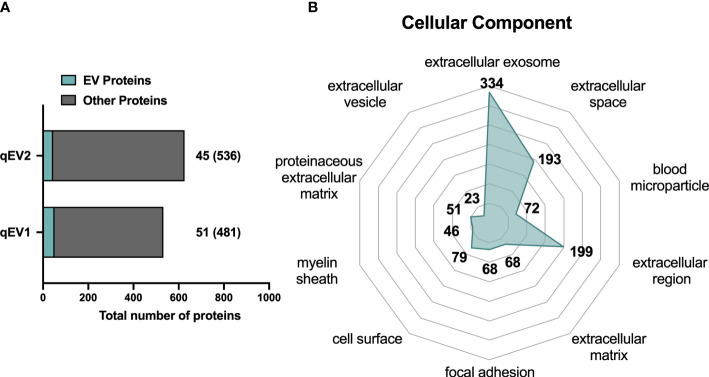
Proteomic analysis of NOD mouse plasma EVs isolated by qEV. Plasma was collected from non-diabetic NOD mice and EVs were enriched using qEV (pooled fractions 7-10). LC-MS/MS analysis was performed with EVs enriched from 800 μL of pooled plasma from two pre-diabetic NOD mice. The two technical replicates were analyzed and are presented as qEV1 and qEV2. **(A)** Bars indicate the total number of proteins identified in the technical replicates. Of these the number of proteins found in the Vesiclepedia top 100 EV protein list are depicted in green. Numbers given in brackets indicate the proteins that are not listed among the top 100 EV proteins. **(B)** The top 10 enriched GO terms for cellular components after GO analysis of all identified proteins. The numbers represent the unique proteins in each term and the enriched GO terms were scored according to a *p*-value <0.05.

Next, we utilized an alternative MS-based strategy (see *Materials and Methods*) to gain a better understanding of the scale of the protein coverage that could be obtained when analyzing EVs enriched from plasma, and to catalogue the protein content of the enriched EVs. We used qEV to isolate EVs by SEC from 800 μl of pooled plasma collected from two pre-diabetic NOD mice and analyzed two technical replicates of the sample (qEV1 and qEV2) by MS. A total of 680 proteins were identified (**Table S1**). We then, cross-referenced the whole list of identified proteins with the online database Vesiclepedia to search for proteins commonly found in EVs. A total of 53 proteins were found in the two qEV replicates that were also among the top 100 proteins previously found in EV samples in other resources (28) (qEV1, n = 51; qEV2, n = 45; **Table S1** and **Figure 3A**). Some classical EVs markers, including the tetraspanins CD9 and CD81, major histocompatibility complex (MHC) class I and II, and heat shock proteins were included in the 53 proteins identified. Moreover, several plasma proteins were detected including immunoglobulins, complement factors, and lipoproteins (**Table S1**).

Finally, all of the proteins included in the list were converted into their respective gene names and were subjected to GO analysis for cellular component terms using the DAVID database. The top 10 enriched terms are represented in **Figure 3B**, and include terms such as extracellular exosome, extracellular space, and blood microparticle.

### 3.5 The plasma EV proteome differs from the whole plasma proteome and contains tissue specific proteins

**Figure 4:**
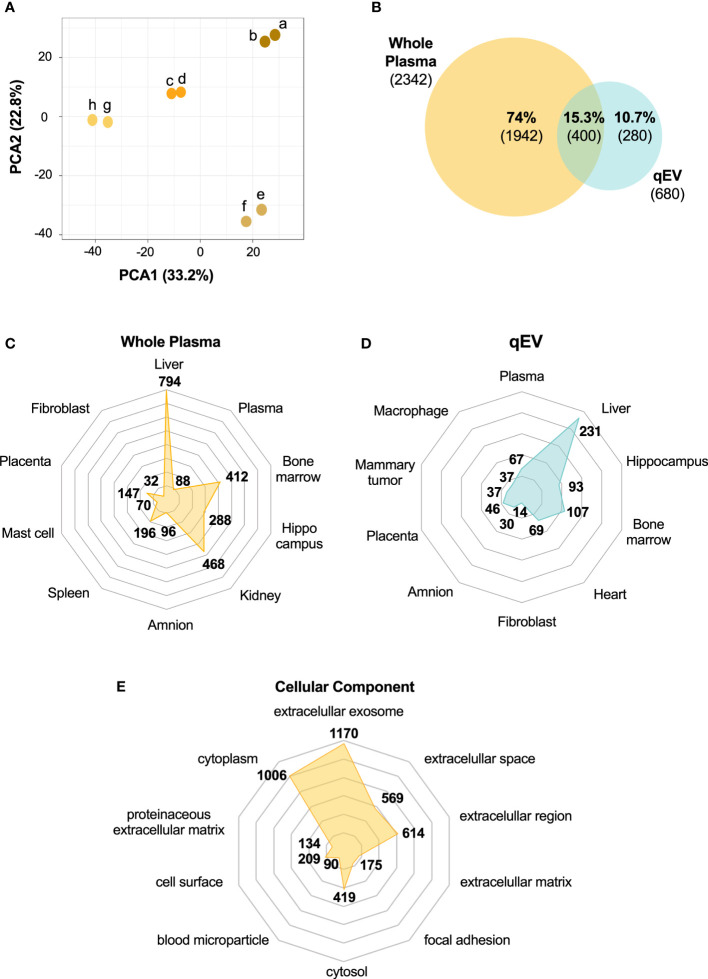
The whole plasma proteome differs from the plasma-derived EV proteome. Plasma was collected from non-diabetic NOD mice (n = 4). Abundant plasma proteins were removed using an affinity column. Samples (two technical replicates of each) were then subjected to HiRIEF-LC-MS/MS analysis. **(A)** Principal Component Analysis (PCA) of the proteome of whole plasma samples showing the clustering of the technical replicates (technical replicates are shown in the same color). **(B)** Venn Diagram of unique and overlapping proteins between whole plasma and qEV proteomes presented as percentages of the total number of proteins identified and absolute numbers. **(C, D)** Tissue enrichment analysis of both proteomes showing the top 10 most significant tissues in whole plasma and qEV samples, respectively. **(E)** Top 10 enriched GO terms for cellular components of the whole plasma proteome. The numbers in C-E represent the counts in each term and the enriched GO terms were scored according to a p-value <0.05.

We next wished to compare the qEV-enriched plasma EV proteome with the proteome of whole plasma samples. For this comparison, new plasma samples were collected and analyzed using a method that enables deeper proteomics coverage than the methods previously used, namely HiRIEF LC-MS/MS. This method is preferentially used for the analyses of complex biological samples including plasma and serum. It includes a pre-analytical step in which the samples are fractionated by high-resolution isoelectric focusing prior to LC-MS/MS (19) and it increases the protein coverage manifold compared to other MS/MS methods (19, 36). Plasma was collected from four prediabetic female NOD mice (10 weeks old) and following the removal of abundant plasma proteins using the affinity removal column (see *Materials and Methods*), the plasma was analyzed using HiRIEF LC-MS/MS. Two technical replicates of each of the four plasma samples were analyzed, and they showed high similarity (**Figure 4A**). Across the whole sample set, a total of 2342 proteins and 19301 peptides were detected (**Table S2**).

Of all the proteins identified in the whole plasma (n = 2342, **Table S2**) and in the EV samples (n = 680, **Table S1**), 15% were found in both of the samples (**Figure 4B**). Around 60% of the proteins detected in the EV samples were also present in the whole plasma samples (**Table S3**), while the remaining 40% were only found in the EV samples (**Figure 4B**, **Table S4**). The conversion of protein to gene names and the subsequent analysis of tissue/cell-enriched gene expression and cellular component (**Figures 4C–E**) using the DAVID database suggested that both the plasma and the EV proteomes contained proteins with tissue- and cell specific origin or enrichment (**Figures 4C, D**). While many of the proteins found in both the whole plasma and qEV samples are enriched in or originate from the liver, there was a greater enrichment of proteins that are expressed in heart tissue in the qEV samples. When searching for terms relevant to T1D, including the pancreas or pancreatic islets, we found that 106 of the whole plasma proteins were classified by the term “pancreas” and included, for example, glucagon and digestive enzymes such as pancreatic lipase, amylase and carboxypeptidase A2 (**Table S5**). In the EV samples a total of 5 proteins with the gene names *Adgrf5, Cpne1, Igfals, Tln1 and Trmp3*, were classified by the term “Adult pancreatic islets”. Using the translational human pancreatic islet genotype tissue-expression resource (TIGER) data portal (23), which integrates RNA-seq data from >400 human pancreatic islets batches with genotype-tissue expression (GTEx) data (37), we investigated the tissue specific expression of the 5 corresponding genes in humans. However, none of the genes were specifically expressed by pancreatic islets, an observation which was confirmed in the Human Protein Atlas (HPA, not shown) (24–26).

Finally, we analyzed in greater detail the list of proteins that were unique to the EV-enriched samples (i.e. proteins not detected in whole plasma samples, **Table S4**). Two of the five genes previously classified with the term “Adult pancreatic islets” (see above), *Cpne1* and *Trpm3*, were found in the list of unique EV proteins. Moreover, by using TIGER we found that several genes which correspond to proteins on the list are highly expressed by islets. The expression of these genes was also found in other tissues to varying degrees, expect for *Atp5j2*. However, according to HPA which integrates both protein expression data and GTEx data, the protein encoded by the gene *Atp5j2* (ATP synthase membrane subunit f) has been classified with “low tissue specificity”. The list of EV-unique proteins also contained proteins with tissue specific expression such as *Sftpd* or with tissue enriched expression such as *Cacna1s* and *Cpne9.*

## 4 Discussion

In the present study, we demonstrated that parallel in-depth proteome analyses of plasma samples and plasma-enriched EVs substantially increase the number of proteins detectable in plasma samples from the NOD mouse. Moreover, we found that the capacity to detect tissue-enriched proteins differs between whole plasma and EV enriched samples, suggesting that the simultaneous analysis of whole plasma and plasma derived EVs facilitates the discovery of such proteins.

Proteins are useful biomarkers thanks to the availability of a broad range of technologies that allow for their identification and quantification in complex biological samples. It has been shown that protein biomarkers are particularly useful in T1D; the appearance of islet autoantibodies marks the initiation of immune-mediated processes that can lead to beta cell destruction and T1D, and C-peptide can be measured to evaluate residual endogenous insulin production. Recently, an increased ratio of proinsulin to insulin/C-peptide has been observed in individuals with T1D or at risk of developing the disease (38). Such a raised ratio also appears to be a predictive marker for an increased risk of future diabetes development among autoantibody positive first-degree relatives (38). Based on these observations and technological advances, it is feasible that additional protein biomarkers can be uncovered which have the potential to provide insight into disease endotypes, have predictive power, or even facilitate the timing of a disease intervention.

The main goal of this study was to develop a protocol for improved detection (with high coverage) of tissue-enriched proteins in plasma samples from an experimental model of T1D, the NOD mouse. We hypothesized that the enrichment of an EV fraction and subsequent proteomics-based analysis (run in parallel with whole plasma) would increase the number of proteins detected by LC-MS/MS. A few studies have reported the enrichment of exosomes or EVs from NOD mouse serum or plasma using ExoQuick, UC or SEC for the purpose of analyzing miRNAs (39, 40)[for a recent review on EVs in T1D, see (13)]. To the best of our knowledge, previous studies have not enriched such samples for subsequent proteomics analysis.

Here we first tested two methods based on different principles for EV enrichment, SEC (qEV) and MA (exoEasy), and examined their ability to enrich EVs from plasma samples collected from NOD mice. Both can be used with the small volumes of plasma which can be collected from one mouse. The average size and range of particle sizes differed when comparing samples enriched using either qEV (factions 7-10) or exoEasy. Particles enriched using exoEasy had a broader size range and larger average size compared to those isolated with qEV, suggesting that to some degree the different methods purify distinctive particle populations. Similar observations regarding differences in particle size have been made when comparing EVs enriched from human plasma samples using the qEV and exoEasy methods (41). The total yield of particles per microliter of plasma was higher when using qEV than exoEasy, and was comparable with or slightly higher than yields that have been previously reported when isolating EVs from mouse plasma (42, 43).

Lipoproteins are small particles (7 – 1200 nm) (44) present at high concentrations in blood and are a common in EV preparations from plasma (31, 32). Our MS analyses demonstrated that apolipoproteins (A-IV, B and E; **Table 1**) were among the 10 most abundant proteins in EV samples enriched by qEV, suggesting that SEC is prone to co-enrich lipoprotein particles. This therefore may explain in part the high particle yield and ratio of particle number/microgram of protein detected using this method. However, EM analysis revealed the presence of vesicles with round or cup-shaped morphology (which are indicative of EVs) in qEV enriched samples while these types of vesicles were less frequently observed in exoEasy-enriched EV samples. The particles found in exoEasy enriched EV samples were large in size (>200nm) with irregular, non-spherical shapes. Such particles were not observed in the qEV samples, however they were previously observed in exoEasy enriched EV samples from human plasma (41), and the authors of this study suggested that they constituted protein aggregates.

We next set out to explore whether a few additional pre-analytical measures could be utilized to increase the protein coverage by LC-MS/MS in whole plasma samples. It is well known that the analysis of plasma proteomes is challenging due to the presence of a few highly abundant proteins which constitute >99% of the total plasma proteome. The removal of such proteins typically expands protein coverage by MS-based methods (33–35). Our initial MS-based analyses showed that abundant plasma proteins were also present in our EV samples. This observation is consistent with numerous other reports [e.g (14, 35)] and moreover, a recent study suggested that a substantial proportion of this plasma protein “contamination” comes from a protein corona that forms spontaneously around EVs circulating in the blood (45).

The presence of abundant plasma proteins was particularly noticeable in the exoEasy (MA) enriched EV samples however after running samples through the depletion column, the abundance of proteins such as albumin was efficiently decreased. As expected, the depletion of the three most abundant plasma proteins from whole plasma samples increased the number of proteins identified by MS. However, after depleting the EV samples no increase in protein coverage detected by MS-analysis was seen. This outcome could for example be due to the complexity of the samples (plasma contains more proteins than EVs) and that the relative abundance of proteins such as albumin is much higher in whole plasma compared to EV enriched samples. Regardless of the reason, the depletion of the abundant plasma proteins did not increase the MS protein coverage in EV samples and, as this type of pre-analytical step is associated with sample loss, we did not further pursue this step before analyzing the EV proteome. Finally, when comparing the proteins identified in EV enriched samples with those in whole plasma, we noted that the whole plasma proteome overlapped more with the proteome of the exoEasy enriched samples than it did with that of the qEV enriched samples. Given that our goal was to identify as many unique proteins as possible from EV fractions, we selected qEV as the most suitable method for further studies.

In addition to the removal of abundant proteins, the reduction in complexity through fractionation of a biofluid such as plasma can facilitate MS-based identification and quantification of low-abundance proteins (19, 36). In the present study, by combining depletion and fractionation we were able to detect more than 2300 proteins in whole plasma samples collected from NOD mice. The HiRIEF LC-MS/MS method used requires more extensive sample preparation and longer analysis time compared to more standard methods. Conversely, this is outweighed by the high protein coverage which is advantageous for the discovery of low abundance proteins in the sample. Consistent with the latter, a previous study which utilized HiRIEF showed that prostate-specific antigen protein (PSA) could be detected when analyzing female plasma samples spiked with PSA at clinically relevant levels (36). New developments in this area including improved pre-analytical protocols and the establishment of semiautomatic workflows (recently reviewed in (11)), should remove bottlenecks and streamline the use of this technology for use in the discovery phase of future biomarker studies.

HiRIEF is particularly useful for highly complex biofluids such as plasma and as EV-enriched plasma fractions have a lower complexity than plasma, HiRIEF analysis of EV samples was not performed in the present study. However, we were able to uncover as many as 680 proteins in qEV-enriched EV samples. When comparing this list of proteins with those detected in whole plasma, we found that around 40% of the proteins identified in the EV enriched samples were not detected in the whole plasma samples. Our GO- and tissue enrichment analyses provided evidence that the EV enriched proteome represents a subfraction of the plasma proteome which is suitable for detecting proteins that cannot be found during the analysis of whole plasma samples. Many of the proteins found in both the whole plasma and qEV samples were enriched in or originated from the liver. Interestingly, there was a greater enrichment of proteins that are expressed in heart tissue in the EV samples than in the whole plasma samples. Whether this is related the way blood was collected (*via* cardiac puncture) remains unknown.

When using TIGER and HPA to examine whether the proteins that were unique to the EV-enriched samples had tissue- or cell specific expression profiles, we found many examples of proteins with tissue enriched expression. These included *Cacna1s* which encodes calcium voltage-gated channel subunit alpha1 S and is primarily expressed in skeletal muscle, cardiomyocytes and the tongue, and *Cpne9*, the Copine family member 9 protein, which has an enriched expression in the brain. Proteins with a tissue specific expression were also found such as surfactant protein D *(Sftpd)*, which is produced by lung alveolar cells. Of note, we also detected the protein TRPM3 (transient receptor potential cation channel subfamily M member 3), which was classified by the term “Adult pancreatic islets”, and according to the Human Protein Atlas has an enriched expression in the brain. TIGER, which integrates GTEx data with large sets of independent human pancreatic islet transcriptome data, suggested however that this gene is also expressed by pancreatic islets, the kidney-cortex and nerves. Previous studies have reported that activation of TRPM3 in pancreatic beta cells enhances insulin secretion (46, 47). These observations suggest that TRPM3 is likely to have originated from a limited number of tissues/cells-types, with the pancreatic beta cell being one of them. Taken together, these observations imply that EV enrichment enables the identification of proteins that are not detectable in whole plasma subjected to MS-based analysis.

In conclusion, this study shows that the parallel proteome profiling of whole plasma samples subjected to the depletion of abundant proteins and peptide fractionation, and of EV-enriched plasma fractions increases the number of plasma proteins which can be uncovered in samples collected from an experimental model of T1D, the NOD mouse. Studies in the NOD mouse have helped to understand the potential mechanisms behind beta-cell destruction and the model has also been used in the development of preventive therapies which have later shown promising outcomes when tested in disease prevention trials (e.g. anti-CD3 treatment) (48, 49). The impact of various environmental factors on the autoimmune process (and particularly diabetes development) have been modelled in the NOD mouse including the effects of antibiotic treatment, microbial exposures, and infections with viruses implicated in human T1D (e.g., Coxsackie B viruses) (6, 9, 50, 51). Furthermore, the effect of certain T1D genetic polymorphisms, molecular mimicry and alterations in beta cell function and immune defense have also been explored in the NOD mouse [e.g (9, 52, 53)] and some of these models may in the future provide relevant tools for understanding T1D disease endotypes. If applied to plasma samples collected from the models described above, the integrated proteome profiling scheme described herein may be useful for the discovery of new, tentatively endotype-specific biomarkers. Coxsackie B virus infections have been linked to the development of T1D in humans (5). As such, one example could be to study plasma samples collected from Coxsackie B virus infected mice prior to diabetes development with a goal to discover biomarkers that are specific for virus-induced beta-cell damage prior to overt disease development. Such protein biomarkers could then be searched for in plasma samples collected in longitudinal studies of individuals at risk of developing T1D using targeted methods, which are less expensive and intended for the analyses of large numbers of samples.

## Data availability statement

The datasets presented in this study can be found in online repositories. The names of the repository/repositories and accession number(s) can be found below: https://www.ebi.ac.uk/pride/archive/, PXD033867.

## Ethics statement 

The animal study was reviewed and approved by Linköpings Försöksdjursetiska Nämnd, Linköping, Sweden.

## Author contributions

MF-T, and SG, conceived the study. IL, HS, ME, MP, SG, and MF-T designed the research. IL, HS, ME, XC, and VS performed experiments/analyses. IL, HS, ME, MP, SG, and MF-T interpreted the results of experiments. IL, HS, XC, and MF-T analyzed the data. IL and HS prepared the figures. MF-T and IL wrote the manuscript with assistance from HS, and VS. HS, ME, XC, MP, and SG reviewed and edited the manuscript. All authors contributed to the article and approved the submitted version.

## Funding

We would like to acknowledge the financial support from the Johnson & Johnson and Karolinska Institutet Partnership program, Karolinska Institutet including the Strategic Research Program in Diabetes, the Swedish Child Diabetes Foundation, the Swedish Diabetes Foundation, the Novo Nordic Foundation, the European Regional Development Fund, the Swedish Medical Research Council and the program Mobilitas Pluss (MOBJD512). We also thank the Karolinska University Hospital for infrastructure support. The funder was not involved in the study design, collection, analysis, interpretation of data, the writing of this article or the decision to submit it for publication.

## Acknowledgments

We would like to thank Dr. Samir El Andaloussi, Karolinska Institutet, Stockholm, Sweden, for critical reading of the manuscript and for giving access to his laboratory Nano Sight NS500 nanoparticle analyzer. We thank Dr. Anirudra Parajuli, Karolinska Institutet, Stockholm, Sweden, for assistance with the PCA analyses.

## Conflict of interest

The authors declare that the research was conducted in the absence of any commercial or financial relationships that could be construed as a potential conflict of interest.

## Publisher’s note

All claims expressed in this article are solely those of the authors and do not necessarily represent those of their affiliated organizations, or those of the publisher, the editors and the reviewers. Any product that may be evaluated in this article, or claim that may be made by its manufacturer, is not guaranteed or endorsed by the publisher.

## Supplementary material

Supplementary Table 6 Gene entries not recognized by DAVID v6.8 using the Functional Enrichment Analysis tool.
