# Supplementary material for: Proteome profiling of whole plasma and plasma-derived extracellular vesicles facilitates the detection of tissue biomarkers in the non-obese diabetic mouse
Source: Front Endocrinol (Lausanne). 2022 Sep 28;13:971313. doi: 10.3389/fendo.2022.971313 (PMC9563222; doi:10.3389/fendo.2022.971313)
Supplement: Supplementary file 1 [file Image_1.pdf]

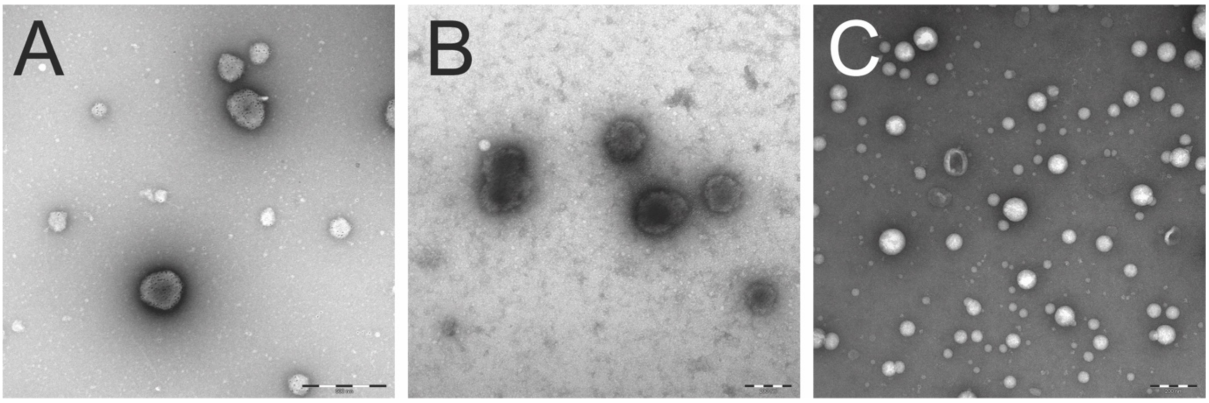

Figure S1. TEM pictures of extracellular vesicle (EV) enriched from plasma using SEC or membrane affinity. Blood samples from pre-diabetic NOD mice were collected in EDTA tubes. Plasma was isolated and filtered through a 0.8 µM filter prior to EV enrichment. EVs were enriched using one of two isolation methods, Size Exclusion Chromatography (SEC) using the qEVoriginal 70 nm column (iZon), or Membrane affinity (MA) using exoEasy Maxi spin columns (Qiagen). (A) TEM picture of EVs enriched using exoEasy. The sample had been subjected to immunogold labeling using an irrelevant antibody (small black dots). Scale bar size 500 nM. (B, C) TEM picture of EVs enriched using qEVoriginal 70 nm column. Scale bars size 200 nM.
