# Supplementary material for: Proteome profiling of whole plasma and plasma-derived extracellular vesicles facilitates the detection of tissue biomarkers in the non-obese diabetic mouse
Source: Front Endocrinol (Lausanne). 2022 Sep 28;13:971313. doi: 10.3389/fendo.2022.971313 (PMC9563222; doi:10.3389/fendo.2022.971313)
Supplement: Supplementary file 2 [file Image_2.pdf]

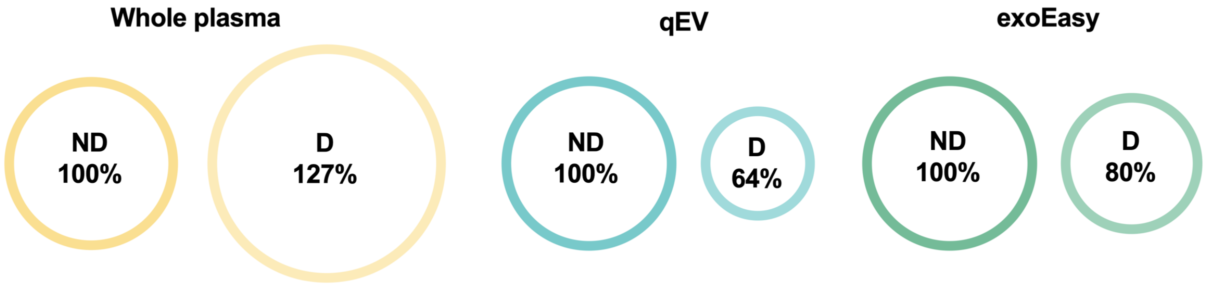


**Figure S2. Successful removal of abundant plasma proteins increases the protein coverage by LC-MS/MS in whole plasma samples but not EV-enriched plasma fractions** Plasma was collected from non-diabetic NOD mice, frozen and pooled before analysis. A whole plasma sample or EV samples enriched using qEV (SEC) or exoEasy (MA) were either untreated or subjected to removal of three high-abundant plasma proteins using the Multiple Affinity Removal Spin Cartridge Mouse-3 column (Agilent Technologies, CA, USA). LC-MS/MS analysis was performed and the effect of the depletion of high abundance proteins (albumin, serotransferrin and IgGs) on the number of proteins identified by MS in the whole plasma, qEV and exoEasy EV samples are shown. Circle size represents the relative increase or decrease in the number of unique proteins uncovered after column depletion compared to no depletion. Percentages were calculated according to number of proteins in each MS run for whole plasma non-depleted (n = 1); whole plasma depleted (n = 2); qEV non-depleted (n = 1); qEV depleted (n = 1); exoEasy non-depleted (n = 2); exoEasy depleted (n = 2). D, depleted; ND, non-depleted.
