# Supplementary material for: Proteome profiling of whole plasma and plasma-derived extracellular vesicles facilitates the detection of tissue biomarkers in the non-obese diabetic mouse
Source: Front Endocrinol (Lausanne). 2022 Sep 28;13:971313. doi: 10.3389/fendo.2022.971313 (PMC9563222; doi:10.3389/fendo.2022.971313)
Supplement: Supplementary file 3 [file DataSheet_1.docx]

**Supplementary table 1.** Proteins identified in NOD mouse plasma EVs enriched by qEV (SEC). Samples were subjected to LC-MS/MS analysis. Proteins listed in the Vesiclepedia top 100 EV protein list are indicated (x).

| Gene Symbol |  | Protein Name | Vesiclepedia Top 100 | | |
| --- | --- | --- | --- | --- | --- |
| 1300017J02Rik |  | RIKEN cDNA 1300017J02 gene | |  |  |
| 2210010C04Rik |  | RIKEN cDNA 2210010C04 gene | |  |  |
| A0A0A0U847 |  | Envelope protein | |  |  |
| A0A0D4DAH6 |  | Immunglobulin light chain variable region (Fragment) | |  |  |
| A0A0D5ZY64 |  | IgE L chain kappa | |  |  |
| A0A1X9HIL1 |  | IgG light chain variable domain (Fragment) | |  |  |
| A0A1X9HIL9 |  | IgG heavy chain variable domain (Fragment) | |  |  |
| A0A2S0DFH5 |  | Anti-HIV fusion peptide monocolonal anitbody light chain vFP5.01 (Fragment) | |  |  |
| A0A2S0DFH6 |  | Anti-HIV fusion peptide monocolonal anitbody light chain vFP1.01 (Fragment) | |  |  |
| A0A2S0DFH8 |  | Anti-HIV fusion peptide monocolonal anitbody light chain vFP6.01 (Fragment) | |  |  |
| A2m |  | Alpha-2-macroglobulin-P | | X |  |
| A2MY50 |  | Anti-acid phosphatase variable light chain 11 (Fragment) | |  |  |
| A2NU19 |  | VH coding region (Fragment) | |  |  |
| A2NVE9 |  | Immnuoglobulin kappa light chain (Fragment) | |  |  |
| A2NW55 |  | E8 variable heavy chain (Fragment) | |  |  |
| Aars2 |  | Alanine--tRNA ligase, mitochondrial | |  |  |
| Abcg2 |  | ATP-binding cassette sub-family G member 2 (Fragment) | |  |  |
| Abi1 |  | Isoform 5 of Abl interactor 1 | |  |  |
| Abi3bp |  | Abi3bp protein (Fragment) | |  |  |
| Acan |  | Aggrecan core protein | |  |  |
| Ace |  | Angiotensin-converting enzyme (Fragment) | |  |  |
| Actb |  | Actin, cytoplasmic 1 | | X |  |
| Actb |  | Actin, cytoplasmic 1 | | X |  |
| Adam10 |  | Disintegrin and metalloproteinase domain-containing protein 10 | |  |  |
| Adgra3 |  | Uncharacterized protein (Fragment) | |  |  |
| Adgre5 |  | Adhesion G protein-coupled receptor E5 | |  |  |
| Adgrf5 |  | Adhesion G protein-coupled receptor F5 (Fragment) | |  |  |
| Adipoq |  | Adiponectin | |  |  |
| Afm |  | Afamin | |  |  |
| Agrn |  | Mini-agrin | |  |  |
| Ahnak |  | AHNAK nucleoprotein (desmoyokin) | |  |  |
| Ahnak2 |  | AHNAK nucleoprotein 2 (Fragment) | |  |  |
| Ahsg |  | Alpha-2-HS-glycoprotein | |  |  |
| Alb |  | Serum albumin | | X |  |
| Aldh16a1 |  | Aldehyde dehydrogenase family 16 member A1 | |  |  |
| Aldh9a1 |  | Gamma-aminobutyraldehyde dehydrogenase | |  |  |
| Aldoa |  | Fructose-bisphosphate aldolase A (Fragment) | | X |  |
| Ambp |  | Protein AMBP | |  |  |
| Amy1 |  | Alpha-amylase 1 | |  |  |
| Angpt1 |  | Angiopoietin-1 | |  |  |
| Angptl3 |  | Angiopoietin-related protein 3 | |  |  |
| Angptl6 Agf |  | Angiopoietin-related protein 6 | |  |  |
| Anpep |  | Aminopeptidase N | |  |  |
| Antxr2 |  | Anthrax toxin receptor 2 | |  |  |
| Anxa1 |  | Annexin A1 | | X |  |
| Anxa11 |  | Annexin A11 | | X |  |
| Anxa2 |  | Annexin A2 (Fragment) | | X |  |
| Anxa3 |  | Annexin A3 (Fragment) | |  |  |
| Anxa4 |  | Annexin A4 | |  |  |
| Anxa5 |  | Annexin A5 | | X |  |
| Anxa6 |  | Annexin A6 | | X |  |
| Anxa7 |  | Annexin A7 | | X |  |
| Apcs |  | Serum amyloid P-component | |  |  |
| Apoa1 |  | Apoa1 protein | |  |  |
| Apoa1 |  | Apolipoprotein A-I | |  |  |
| Apoa2 |  | Apolipoprotein A-II | |  |  |
| Apoa4 |  | Apolipoprotein A-IV | |  |  |
| Apoa4 |  | Apolipoprotein A-IV | |  |  |
| Apoa5 |  | Apolipoprotein A-V | |  |  |
| Apob |  | Apolipoprotein B-100 | |  |  |
| Apoc1 |  | Apolipoprotein C-I | |  |  |
| Apoc2 |  | Apolipoprotein C-II | |  |  |
| Apoc3 |  | Apolipoprotein C-III | |  |  |
| Apoc4 |  | Apolipoprotein C-IV | |  |  |
| Apod |  | Apolipoprotein D | |  |  |
| Apoe |  | Apolipoprotein e | |  |  |
| Apoe |  | Apolipoprotein e | |  |  |
| Apoh |  | Beta-2-glycoprotein 1 | |  |  |
| Apom |  | Apolipoprotein M | |  |  |
| Apon |  | Apolipoprotein N | |  |  |
| App |  | Amyloid-beta A4 protein (Fragment) | |  |  |
| Aqp1 |  | Aquaporin-1 | |  |  |
| Arf3 |  | ADP-ribosylation factor 3 | |  |  |
| Arhgdia |  | Rho GDP-dissociation inhibitor 1 | |  |  |
| Arhgdib |  | Rho GDP-dissociation inhibitor 2 | |  |  |
| Arpc4 |  | Actin-related protein 2/3 complex subunit 4 | |  |  |
| Atp1a1 |  | Sodium/potassium-transporting ATPase subunit alpha-1 | | X |  |
| Atp1b1 |  | Sodium/potassium-transporting ATPase subunit beta (Fragment) | |  |  |
| Atp2a2 |  | Isoform 2 of Sarcoplasmic/endoplasmic reticulum calcium ATPase 2 | |  |  |
| Atp2b4 |  | Uncharacterized protein (Fragment) | |  |  |
| Atp5c1 |  | ATP synthase, H+-transporting, mitochondrial F1 complex, gamma polypeptide 1 (Fragment) | |  |  |
| Atp5f1a |  | ATP synthase subunit alpha, mitochondrial | |  |  |
| Atp5f1b |  | ATP synthase subunit beta, mitochondrial | |  |  |
| Atp5h |  | ATP synthase subunit d, mitochondrial (Fragment) | |  |  |
| Atp5j2 |  | ATP synthase subunit f, mitochondrial | |  |  |
| Atp5o |  | ATP synthase subunit O, mitochondrial (Fragment) | |  |  |
| B2CSK2 |  | Heat shock protein 1-like protein | |  |  |
| B2m |  | Beta-2-microglobulin | |  |  |
| Bag2 |  | BAG family molecular chaperone regulator 2 | |  |  |
| Bche |  | Cholinesterase | |  |  |
| Bcl11b |  | B-cell lymphoma/leukemia 11B | |  |  |
| Bgn |  | Biglycan | |  |  |
| Blvrb |  | Flavin reductase (NADPH) | |  |  |
| Bpifa2 |  | BPI fold-containing family A member 2 (Fragment) | |  |  |
| Bsg |  | Basigin (Fragment) | | X |  |
| Btnl10 |  | Isoform 2 of Butyrophilin-like protein 10 | |  |  |
| C1qa |  | Complement C1q subcomponent subunit A | |  |  |
| C1qb |  | Complement C1q subcomponent subunit B | |  |  |
| C1qc |  | Complement C1q subcomponent subunit C | |  |  |
| C1qtnf9 |  | Isoform 2 of Complement C1q and tumor necrosis factor-related protein 9 | |  |  |
| C1ra |  | Complement component 1, r | |  |  |
| C1s1 |  | Complement component 1, s subcomponent | |  |  |
| C3 |  | Complement C3 | | X |  |
| C4b |  | Complement component 4B (Childo blood group) | |  |  |
| C4bp |  | C4b-binding protein alpha-chain | |  |  |
| C6 |  | Complement component 6 | |  |  |
| C7 |  | Complement component 7 | |  |  |
| C8a |  | Complement component C8 alpha chain | |  |  |
| C8b |  | Complement component C8 beta chain | |  |  |
| C8g |  | Complement component 8, gamma subunit, isoform CRA_b | |  |  |
| C9 |  | Complement component C9 | |  |  |
| Ca2 |  | Carbonic anhydrase 2 | |  |  |
| Cacna1s |  | Voltage-dependent L-type calcium channel subunit alpha | |  |  |
| Cacna2d1 |  | Isoform 2C of Voltage-dependent calcium channel subunit alpha-2/delta-1 | |  |  |
| Calm5 |  | Calm5 protein | |  |  |
| Calr |  | Uncharacterized protein (Fragment) | |  |  |
| Cap1 |  | Adenylyl cyclase-associated protein 1 (Fragment) | | X |  |
| Caprin2 |  | Caprin-2 | |  |  |
| Capza1 |  | F-actin-capping protein subunit alpha-1 (Fragment) | |  |  |
| Capzb |  | F-actin-capping protein subunit beta (Fragment) | |  |  |
| Casq2 |  | Calsequestrin (Fragment) | |  |  |
| Cat |  | Catalase | |  |  |
| Cav1 |  | Caveolin-1 | |  |  |
| Cavin1 |  | Caveolae-associated protein 1 | |  |  |
| Cchcr1 |  | Coiled-coil alpha-helical rod protein 1 (Alpha-helical coiled-coil rod protein) | |  |  |
| Ccp110 |  | Centriolar coiled-coil protein of 110 kDa | |  |  |
| Cd200 |  | OX-2 membrane glycoprotein | |  |  |
| Cd3e |  | CD3 antigen epsilon polypeptide | |  |  |
| Cd44 |  | CD44 antigen | |  |  |
| Cd47 |  | Leukocyte surface antigen CD47 | |  |  |
| Cd5 |  | T-cell surface glycoprotein CD5 | | X |  |
| Cd5l |  | CD5 antigen-like | |  |  |
| Cd81 |  | CD81 antigen (Fragment) | | X |  |
| Cd9 |  | CD9 antigen | |  |  |
| Cdc42 |  | Cell division control protein 42 homolog | | X |  |
| Cdh1 |  | Cadherin 1, isoform CRA_b | |  |  |
| Cdh5 |  | Cadherin-5 | |  |  |
| Cenpf |  | Centromere protein F (Fragment) | |  |  |
| Cep162 |  | Uncharacterized protein | |  |  |
| Cep55 |  | Isoform 2 of Centrosomal protein of 55 kDa | |  |  |
| Ces1b |  | Carboxylic ester hydrolase | |  |  |
| Ces1c |  | Carboxylesterase 1C | |  |  |
| Ces2e |  | Pyrethroid hydrolase Ces2e | |  |  |
| Ces3a |  | Isoform 2 of Carboxylesterase 3A | |  |  |
| Cfb |  | Complement factor B | |  |  |
| Cfh |  | Complement factor H | |  |  |
| Cfi |  | Complement factor I | |  |  |
| Cfl1 |  | Cofilin-1 | | X |  |
| Cfp |  | Properdin | |  |  |
| Cilp |  | Cartilage intermediate layer protein 1 | |  |  |
| Ckmt2 |  | Creatine kinase S-type, mitochondrial | |  |  |
| Clca3a2 |  | Chloride channel accessory 3A2 (Fragment) | |  |  |
| Clec11a |  | C-type lectin domain family 11 member A | |  |  |
| Clec1b |  | Isoform 2 of C-type lectin domain family 1 member B | |  |  |
| Clec4f |  | C-type lectin domain family 4 member F | |  |  |
| Clic4 |  | Chloride intracellular channel protein 4 | |  |  |
| Cltc |  | Clathrin heavy chain 1 | | X |  |
| Clu |  | Clusterin | |  |  |
| Col12a1 |  | Collagen alpha-1(XII) chain | |  |  |
| Col18a1 |  | Isoform 3 of Collagen alpha-1(XVIII) chain | |  |  |
| Col1a1 |  | Isoform 2 of Collagen alpha-1(I) chain | |  |  |
| Col1a2 |  | Collagen alpha-2(I) chain | |  |  |
| Col2a1 |  | Isoform 3 of Collagen alpha-1(II) chain | |  |  |
| Col3a1 |  | Collagen alpha-1(III) chain (Fragment) | |  |  |
| Col4a2 |  | Collagen alpha-2(IV) chain | |  |  |
| Col6a1 |  | Collagen alpha-1(VI) chain | |  |  |
| Col6a2 |  | Collagen alpha-2(VI) | |  |  |
| Col6a3 |  | Collagen, type VI, alpha 3 | |  |  |
| Col6a5 |  | Collagen alpha-5(VI) chain | |  |  |
| Colec10 |  | Collectin-10 | |  |  |
| Colec11 |  | Collectin-11 | |  |  |
| Comp |  | Cartilage oligomeric matrix protein | |  |  |
| Coro1a |  | Coronin-1A | |  |  |
| Cotl1 |  | Coactosin-like protein | |  |  |
| COX2 |  | Cytochrome c oxidase subunit 2 | |  |  |
| Cox4i1 |  | Cytochrome c oxidase subunit 4 isoform 1, mitochondrial | |  |  |
| Cox6b1 |  | Cytochrome c oxidase subunit 6B1 | |  |  |
| Cox7a1 |  | Cytochrome c oxidase subunit 7A1, mitochondrial | |  |  |
| Cp |  | Ceruloplasmin | |  |  |
| Cpn1 |  | Carboxypeptidase N catalytic chain | |  |  |
| Cpn2 |  | Carboxypeptidase N subunit 2 | |  |  |
| Cpne1 |  | Copine-1 (Fragment) | |  |  |
| Cpne9 |  | MKIAA4217 protein (Fragment) | |  |  |
| Cpt1b |  | Carnitine O-palmitoyltransferase 1, muscle isoform, CPT1-M, EC 2.3.1.21 | |  |  |
| Cramp |  | Cathelicidin (Fragment) | |  |  |
| Cryab |  | Alpha-crystallin B chain (Fragment) | |  |  |
| Csf1r |  | Macrophage colony-stimulating factor 1 receptor | |  |  |
| Cst3 |  | Cystatin (Fragment) | |  |  |
| Cst6 |  | Cystatin (Fragment) | |  |  |
| Ctsb |  | Cathepsin B | |  |  |
| Ctsd |  | Ctsd protein | |  |  |
| Cybb |  | Cytochrome b-245 heavy chain (Fragment) | |  |  |
| Cyc1 |  | Cytochrome c1, heme protein, mitochondrial (Fragment) | |  |  |
| Cycs |  | Cytochrome c, somatic | |  |  |
| Daam1 |  | Isoform 3 of Disheveled-associated activator of morphogenesis 1 | |  |  |
| Dido1 |  | Death-inducer obliterator 1 | |  |  |
| Dlat |  | Dihydrolipoyllysine-residue acetyltransferase component of pyruvate dehydrogenase complex, mitochondrial | |  |  |
| Dlst |  | Dihydrolipoyllysine-residue succinyltransferase component of 2-oxoglutarate dehydrogenase complex, mitochondrial | |  |  |
| Dmkn |  | Dermokine | |  |  |
| Dnajc5 |  | DnaJ homolog subfamily C member 5 (Fragment) | |  |  |
| DOM-7 |  | Serpina1 DOM-7 | |  |  |
| Dpep1 |  | Dipeptidase 1 | |  |  |
| Dspp |  | Dentin sialophosphoprotein | |  |  |
| Ecm1 |  | Extracellular matrix protein 1 (Fragment) | |  |  |
| Eea1 |  | Eea1 protein (Fragment) | |  |  |
| Egfr |  | Epidermal growth factor receptor isoform 2 | |  |  |
| Ehd2 |  | EH domain-containing protein 2 | |  |  |
| Ehd4 |  | EH domain-containing protein 4 (PAST homolog 2, mPAST2) | |  |  |
| Emilin1 |  | Elastin microfibril interfacer 1 (Fragment) | |  |  |
| Eng |  | Endoglin (Fragment) | |  |  |
| Eno1 |  | Alpha-enolase | | X |  |
| Entpd1 |  | Ectonucleoside triphosphate diphosphohydrolase 1 (Fragment) | |  |  |
| Epb41 |  | Protein 4.1 (Fragment) | |  |  |
| Esd |  | S-formylglutathione hydrolase (Fragment) | |  |  |
| Etfdh |  | Electron transfer flavoprotein-ubiquinone oxidoreductase, mitochondrial | |  |  |
| Ezr |  | Ezrin (Fragment) | | X |  |
| F10 |  | Coagulation factor X | |  |  |
| F13a1 |  | Coagulation factor XIII A chain | |  |  |
| F13b |  | Coagulation factor XIII B chain | |  |  |
| F2 |  | Prothrombin | |  |  |
| F5 |  | Coagulation factor V | |  |  |
| Fabp1 |  | Fatty acid-binding protein, liver | |  |  |
| Fam104a |  | Family with sequence similarity 104, member A | |  |  |
| Fap |  | Isoform 3 of Prolyl endopeptidase FAP | |  |  |
| Fbln1 |  | Fibulin-1 | |  |  |
| Fcgbp |  | Fc fragment of IgG-binding protein | |  |  |
| Fcn1 |  | Ficolin-1 | |  |  |
| Fermt3 |  | Fermitin family homolog 3 | |  |  |
| Fetub |  | Fetuin-B | |  |  |
| Fga |  | Fibrinogen alpha chain | |  |  |
| Fgb |  | Fibrinogen beta chain | |  |  |
| Fgg |  | Fibrinogen gamma chain | |  |  |
| Flna |  | Filamin, alpha | | X |  |
| Flt4 |  | Vascular endothelial growth factor receptor 3 | |  |  |
| Fn1 |  | Fibronectin | | X |  |
| Fn1 |  | Fibronectin | | X |  |
| Fn1 |  | Fibronectin (Fragment) | | X |  |
| Fyb1 |  | Isoform FYB-120 of FYN-binding protein 1] | |  |  |
| G0YP42 |  | Anti-human Langerin 2G3 lambda chain | |  |  |
| Ganab |  | Neutral alpha-glucosidase AB | |  |  |
| Gc |  | Vitamin D-binding protein | |  |  |
| Gdi2 |  | Rab GDP dissociation inhibitor (Fragment) | |  |  |
| Glul |  | Glutamine synthetase | |  |  |
| Gm10481 |  | Gp_dh_C domain-containing protein (Fragment) | |  |  |
| Gm10696 |  | Speckle-type BTB/POZ protein family member 2 | |  |  |
| Gm11214 |  | Uncharacterized protein (Fragment) | |  |  |
| Gm38417 |  | MHC class I antigen | |  |  |
| Gm42543 |  | Uncharacterized protein (Fragment) | |  |  |
| Gm5478 |  | Predicted pseudogene 5478 | |  |  |
| Gm7298 |  | Predicted gene 7298 | |  |  |
| Gmnn |  | Geminin (Fragment) | |  |  |
| Gnai2 |  | Guanine nucleotide-binding protein G(i) subunit alpha-2 (Fragment) | | X |  |
| Gnb1 |  | Guanine nucleotide-binding protein G(I)/G(S)/G(T) subunit beta-1 (Fragment) | | X |  |
| Gp1ba |  | Platelet glycoprotein Ib alpha chain] | |  |  |
| Gp1bb |  | Platelet glycoprotein Ib beta chain | |  |  |
| Gp5 |  | Platelet glycoprotein V | |  |  |
| Gpld1 |  | Phosphatidylinositol-glycan-specific phospholipase D | |  |  |
| Gpx3 |  | Glutathione peroxidase 3 | |  |  |
| Gsn |  | Gelsolin | | X |  |
| Gstp1 |  | Glutathione S-transferase P 1 | |  |  |
| Guca1a |  | Guanylyl cyclase-activating protein 1 | |  |  |
| Gucy2c |  | Guanylyl cyclase C (Fragment) | |  |  |
| H2-Aa |  | MHC class II antigen A alpha isoform-004 | |  |  |
| H2-Bl |  | Truncated MHC class I antigen splice variant Bl.2a | |  |  |
| H2-D1 |  | H-2D cell surface glycoprotein (Fragment) | |  |  |
| H2-D1 |  | MHC class I heavy chain maturation peptide H-2D(D) (Fragment) | |  |  |
| H2-K1 |  | MHC class I heavy chain maturation peptide H-2K(D) (Fragment) | |  |  |
| H2-Q10 |  | H-2 class I histocompatibility antigen, Q10 alpha chain | |  |  |
| H2-Q2 |  | MHC class I like protein GS8 (Fragment) | |  |  |
| H2-Q8 |  | H-2 class I histocompatibility antigen, Q8 alpha chain | |  |  |
| H2-T23 |  | MHC class Ib antigen Qa-1c | |  |  |
| H2aj |  | Histone H2A | |  |  |
| H4c1 |  | Histone H4 | | X |  |
| H6pd |  | H6pd protein (Fragment) | |  |  |
| Hadha |  | Trifunctional enzyme subunit alpha, mitochondrial | |  |  |
| Hadhb |  | Trifunctional enzyme subunit beta, mitochondrial | |  |  |
| Hapln1 |  | Hyaluronan and proteoglycan link protein 1 | |  |  |
| Hba |  | Hemoglobin subunit alpha | |  |  |
| Hbat1 |  | Alpha-globin | |  |  |
| Hbb-b2 |  | Beta-2-globin (Fragment) | |  |  |
| Hbb-bs |  | Beta-globin | |  |  |
| HC |  | MAb 110 heavy chain | |  |  |
| HC |  | MAb 31C6 heavy chain | |  |  |
| HC |  | MAb 44B1 heavy chain | |  |  |
| Heg1 |  | Isoform 2 of Protein HEG homolog 1 | |  |  |
| Hgfac |  | Hepatocyte growth factor activator, HGF activator, HGFA, EC 3.4.21.- [Cleaved into: Hepatocyte growth factor activator short chain; Hepatocyte growth factor activator long chain] | |  |  |
| Hist2h2bb |  | Hist2h2bb protein | |  |  |
| Hmcn1 |  | Isoform 2 of Hemicentin-1 | |  |  |
| Hmcn2 |  | Hemicentin-2 | |  |  |
| Hpx |  | Hemopexin | |  |  |
| Hrc |  | Hrc protein | |  |  |
| Hrg |  | Histidine-rich glycoprotein | |  |  |
| Hsp90ab1 |  | Heat shock protein HSP 90-beta | |  |  |
| Hspa5 |  | Endoplasmic reticulum chaperone BiP | | X |  |
| Hspa8 |  | Heat shock cognate 71 kDa protein | | X |  |
| Hspb1 |  | Heat shock protein beta-1 | |  |  |
| Hspg2 |  | Heparan sulfate proteoglycan 2 | |  |  |
| HVM27 |  | Ig heavy chain V-III region A4 | |  |  |
| HVM51 |  | Ig heavy chain V region AC38 205.12 | |  |  |
| HVM57 |  | Ig heavy chain V region 6.96 | |  |  |
| I6L9E1 |  | Uncharacterized protein | |  |  |
| Icam1 |  | Uncharacterized protein (Fragment) | |  |  |
| Igfals |  | Insulin-like growth factor-binding protein complex acid labile subunit] | |  |  |
| Igh |  | Igh protein | |  |  |
| Igh |  | Igh protein | |  |  |
| Igh |  | Igh protein | |  |  |
| Igh |  | Igh protein | |  |  |
| Igh-VJ558 |  | Igh protein | |  |  |
| IGHG3 |  | Isoform 2 of Ig gamma-3 chain C region | |  |  |
| Ighm |  | Immunoglobulin heavy constant mu | |  |  |
| Ighm |  | Immunoglobulin heavy constant mu (Fragment) | |  |  |
| IGHV |  | Immunoglobulin heavy chain variable region (Fragment) | |  |  |
| Ighv1-11 |  | Immunoglobulin heavy variable V1-11 (Fragment) | |  |  |
| Ighv1-12 |  | Immunoglobulin heavy variable V1-12 | |  |  |
| Ighv1-18 |  | Immunoglobulin heavy variable V1-18 (Fragment) | |  |  |
| Ighv1-34 |  | Immunoglobulin heavy variable 1-34 (Fragment) | |  |  |
| Ighv1-42 |  | Immunoglobulin heavy variable V1-42 | |  |  |
| Ighv1-43 |  | Immunoglobulin heavy variable V1-43 | |  |  |
| Ighv1-49 |  | Immunoglobulin heavy variable 1-49 | |  |  |
| Ighv1-62-2 |  | Immunoglobulin heavy variable 1-62-2 | |  |  |
| Ighv1-76 |  | Immunoglobulin heavy variable 1-76 | |  |  |
| Ighv1-78 |  | Immunoglobulin heavy variable 1-78 | |  |  |
| Ighv1-82 |  | Immunoglobulin heavy variable 1-82 | |  |  |
| Ighv10-3 |  | Immunoglobulin heavy variable V10-3 (Fragment) | |  |  |
| Ighv11-1 |  | Immunoglobulin heavy variable 11-1 (Fragment) | |  |  |
| Ighv14-1 |  | Immunoglobulin heavy variable 14-1 (Fragment) | |  |  |
| Ighv14-4 |  | Immunoglobulin heavy variable 14-4 | |  |  |
| Ighv4-1 |  | Immunoglobulin heavy variable 4-1 (Fragment) | |  |  |
| Ighv5-12 |  | Immunoglobulin heavy variable 5-12 (Fragment) | |  |  |
| Ighv5-16 |  | Immunoglobulin heavy variable 5-16 | |  |  |
| Ighv6-3 |  | Immunoglobulin heavy variable 6-3 (Fragment) | |  |  |
| Ighv6-5 |  | Immunoglobulin heavy variable V6-5 | |  |  |
| Ighv7-4 |  | Immunoglobulin heavy variable 7-4 | |  |  |
| Ighv8-12 |  | Immunoglobulin heavy variable V8-12 (Fragment) | |  |  |
| Ighv8-8 |  | Immunoglobulin heavy variable 8-8 (Fragment) | |  |  |
| Ighv9-1 |  | Immunoglobulin heavy variable 9-1 | |  |  |
| Ighv9-3 |  | Immunoglobulin heavy variable V9-3 (Fragment) | |  |  |
| Ighv9-4 |  | Immunoglobulin heavy variable 9-4 | |  |  |
| Igk |  | Igk protein | |  |  |
| Igk |  | Igk protein | |  |  |
| Igkv1-132 |  | Immunoglobulin kappa variable 1-132 (Fragment) | |  |  |
| Igkv12-38 |  | Immunoglobulin kappa chain variable 12-38 | |  |  |
| Igkv12-98 |  | Immunoglobulin kappa variable 12-98 | |  |  |
| Igkv14-130 |  | Immunoglobulin kappa variable 14-130 | |  |  |
| Igkv4-53 |  | Immunoglobulin kappa variable 4-53 | |  |  |
| Igkv4-61 |  | Immunoglobulin kappa chain variable 4-61 | |  |  |
| Igkv4-63 |  | Immunoglobulin kappa variable 4-63 | |  |  |
| Igkv4-80 |  | Immunoglobulin kappa variable 4-80 (Fragment) | |  |  |
| Igkv4-81 |  | Immunoglobulin kappa variable 4-81 | |  |  |
| Igkv5-39 |  | Immunoglobulin kappa variable 5-39 | |  |  |
| Igkv5-43 |  | Immunoglobulin kappa chain variable 5-43 (Fragment) | |  |  |
| Igkv5-45 |  | Immunoglobulin kappa chain variable 5-45 (Fragment) | |  |  |
| Igkv6-15 |  | Immunoglobulin kappa variable 6-15 (Fragment) | |  |  |
| Igkv6-20 |  | Immunoglobulin kappa variable 6-20 (Fragment) | |  |  |
| Igkv8-19 |  | Immunoglobulin kappa variable 8-19 | |  |  |
| Igkv9-123 |  | Immunoglobulin kappa variable 9-123 (Fragment) | |  |  |
| Igkv9-124 |  | Immunoglobulin kappa chain variable 9-124 | |  |  |
| Iglc2 |  | Ig lambda-2 chain C region | |  |  |
| Iglc3 |  | Ig lambda-3 chain C region | |  |  |
| Iglv1 |  | A6 anti-[4-hydroxy-3-nitrophenyl(Phenolate form)] acetyl mAb V-L region (Fragment) | |  |  |
| Iglv3 |  | Immunoglobulin lambda variable 3 (Fragment) | |  |  |
| Il1rap |  | Interleukin-1 receptor accessory protein | |  |  |
| Ilk |  | Integrin-linked protein kinase | |  |  |
| Itga2 |  | Integrin alpha-2 | |  |  |
| Itga2b |  | Integrin alpha 2b | |  |  |
| Itga6 |  | Isoform Alpha-6X1A of Integrin alpha-6 | |  |  |
| Itgal |  | Integrin alpha-L | |  |  |
| Itgb1 |  | Integrin beta-1 | | X |  |
| Itgb2 |  | Integrin beta | |  |  |
| Itgb3 |  | Integrin beta-3 | |  |  |
| Itih1 |  | Inter-alpha trypsin inhibitor, heavy chain 1 (Fragment) | |  |  |
| Itih2 |  | Inter-alpha-trypsin inhibitor heavy chain H2 | |  |  |
| Itih3 |  | Inter-alpha-trypsin inhibitor heavy chain H3 | |  |  |
| Itih4 |  | Inter alpha-trypsin inhibitor, heavy chain 4 | |  |  |
| Jchain |  | Immunoglobulin J chain | |  |  |
| K7T9R7 |  | IgM heavy chain variable region (Fragment) | |  |  |
| K7TH39 |  | IgM heavy chain variable region (Fragment) | |  |  |
| K7THE6 |  | IgM heavy chain variable region (Fragment) | |  |  |
| K7THH3 |  | IgM heavy chain variable region (Fragment) | |  |  |
| K7TRL3 |  | IgA heavy chain variable region (Fragment) | |  |  |
| Klkb1 |  | Plasma kallikrein | |  |  |
| Kng1 |  | Kininogen-1 | |  |  |
| Krt1 |  | Keratin, type II cytoskeletal 1 | | X |  |
| Krt10 |  | Isoform 3 of Keratin, type I cytoskeletal 10 | | X |  |
| Krt14 |  | Keratin, type I cytoskeletal 14 | | X |  |
| Krt16 |  | Keratin, type I cytoskeletal 16 | | X |  |
| Krt2 |  | Krt2 protein | |  |  |
| Krt25 |  | Keratin, type I cytoskeletal 25 | |  |  |
| Krt42 |  | Keratin, type I cytoskeletal 42 | | X |  |
| Krt73 |  | Keratin, type II cytoskeletal 73 | |  |  |
| Krt78 |  | Krt78 protein (Fragment) | |  |  |
| Krt79 |  | Keratin, type II cytoskeletal 79 | |  |  |
| KV2A7 |  | Ig kappa chain V-II region 26-10 | |  |  |
| KV3AG |  | Ig kappa chain V-III region PC 7210 | |  |  |
| KV3AL |  | Ig kappa chain V-III region PC 2485/PC 4039 | |  |  |
| KV5A4 |  | Ig kappa chain V-V region MOPC 149 | |  |  |
| KV5AE |  | Ig kappa chain V-V region HP 124E1 | |  |  |
| KV6A1 |  | Ig kappa chain V-VI region XRPC 44 | |  |  |
| KV6A8 |  | Ig kappa chain V-VI region NQ5-61.1.2 | |  |  |
| KVM5 |  | Ig kappa chain V region Mem5 (Fragment) | |  |  |
| L1cam |  | Neural cell adhesion molecule L1 | |  |  |
| Lama1 |  | Laminin subunit alpha-1 | |  |  |
| Lama2 |  | Laminin subunit alpha-2 | |  |  |
| Lamb1 |  | Laminin subunit beta-1 | |  |  |
| Lamb2 |  | Laminin subunit beta-2 | |  |  |
| Lamc1 |  | Laminin subunit gamma-1 | |  |  |
| Lbp |  | Lipopolysaccharide-binding protein | |  |  |
| LC |  | MAb 6H10 light chain | |  |  |
| Lcat |  | Phosphatidylcholine-sterol acyltransferase | |  |  |
| Ldha |  | L-lactate dehydrogenase (Fragment) | | X |  |
| Ldlr |  | Low-density lipoprotein receptor | |  |  |
| Lgals3bp |  | Galectin-3-binding protein | | X |  |
| Lifr |  | Isoform 2 of Leukemia inhibitory factor receptor | |  |  |
| Lipc |  | Hepatic triacylglycerol lipase | |  |  |
| LOC207685 |  | LOC207685 protein (Fragment) | |  |  |
| Loxl1 |  | Lysyl oxidase homolog 1 | |  |  |
| Lrp1 |  | Low density lipoprotein receptor-related protein 1 | |  |  |
| Lrriq4 |  | Isoform 2 of Leucine-rich repeat and IQ domain-containing protein 4 | |  |  |
| Ltbp1 |  | Latent-transforming growth factor beta-binding protein 1 (Fragment) | |  |  |
| Lum |  | Lumican | |  |  |
| Lyn |  | Tyrosine-protein kinase | |  |  |
| Lyz1 |  | Lysozyme C-1 | |  |  |
| M6pr |  | Cation-dependent mannose-6-phosphate receptor | |  |  |
| Mad1l1 |  | Isoform 2 of Mitotic spindle assembly checkpoint protein MAD1 | |  |  |
| Man1a |  | alpha-1,2-Mannosidase | |  |  |
| Masp1 |  | Isoform 2 of Mannan-binding lectin serine protease 1 | |  |  |
| Masp1 |  | Mannan-binding lectin serine protease 1 | |  |  |
| Masp2 |  | Mannan-binding lectin serine protease 2 | |  |  |
| Mb |  | Myoglobin (Fragment) | |  |  |
| Mbl1 |  | Mannose-binding protein A | |  |  |
| Mbl2 |  | Mannose-binding protein C | |  |  |
| Med14 |  | Isoform 4 of Mediator of RNA polymerase II transcription subunit 14 | |  |  |
| Mfge8 |  | Milk fat globule-EGF factor 8 protein, isoform CRA_a | |  |  |
| Mfsd4b5 |  | Major facilitator superfamily domain-containing 4B5 | |  |  |
| Mgam |  | Maltase-glucoamylase | |  |  |
| Mgam |  | Maltase-glucoamylase | |  |  |
| Mmrn1 |  | Multimerin-1 | |  |  |
| Msn |  | Moesin | | X |  |
| Muc16 |  | Mucin 16 (Fragment) | |  |  |
| Mug1 |  | Murinoglobulin-1 | |  |  |
| Mup14 |  | Major urinary protein 14 (Fragment) | |  |  |
| Mus81 |  | Crossover junction endonuclease MUS81 (Fragment) | |  |  |
| Mvp |  | Major vault protein | |  |  |
| Myh3 |  | Myosin-3 | |  |  |
| Myh6 |  | Myosin-6 | |  |  |
| Myh9 |  | Myosin-9 | | X |  |
| Myl12a |  | 2900073G15Rik protein (Fragment) | |  |  |
| Myl2 |  | Myosin regulatory light chain 2, ventricular/cardiac muscle isoform | |  |  |
| Myl3 |  | Myosin light chain 3 (Fragment) | |  |  |
| Myo1g |  | Unconventional myosin-Ig | |  |  |
| Myo6 |  | Unconventional myosin-VI | |  |  |
| Napsa |  | Napsin-A | |  |  |
| Ndufa2 |  | NADH dehydrogenase [ubiquinone] 1 alpha subcomplex subunit 2 | |  |  |
| Ndufa4 |  | Cytochrome c oxidase subunit NDUFA4 | |  |  |
| Ndufa8 |  | NADH dehydrogenase [ubiquinone] 1 alpha subcomplex subunit 8 | |  |  |
| Ndufa9 |  | NADH dehydrogenase [ubiquinone] 1 alpha subcomplex subunit 9, mitochondrial | |  |  |
| Ndufs1 |  | NADH-ubiquinone oxidoreductase 75 kDa subunit, mitochondrial | |  |  |
| Ndufs2 |  | NADH dehydrogenase [ubiquinone] iron-sulfur protein 2, mitochondrial (Fragment) | |  |  |
| Ndufs3 |  | NADH dehydrogenase [ubiquinone] iron-sulfur protein 3, mitochondrial | |  |  |
| Ndufs7 |  | NADH dehydrogenase [ubiquinone] iron-sulfur protein 7, mitochondrial | |  |  |
| Ndufv1 |  | NADH dehydrogenase [ubiquinone] flavoprotein 1, mitochondrial | |  |  |
| Ndufv2 |  | Isoform 2 of NADH dehydrogenase [ubiquinone] flavoprotein 2, mitochondrial | |  |  |
| Nid1 |  | Nidogen-1 | |  |  |
| Nid2 |  | Nidogen-2 (Fragment) | |  |  |
| Nme1 |  | Nucleoside diphosphate kinase (Fragment) | |  |  |
| Nras |  | GTPase NRas (Fragment) | |  |  |
| Nuak2 |  | Isoform 2 of NUAK family SNF1-like kinase 2 | |  |  |
| Nucb1 |  | Nucleobindin 1, isoform CRA_b | |  |  |
| O19464 |  | MHC class II A polypeptide (Fragment) | |  |  |
| Oit3 |  | Oncoprotein-induced transcript 3 protein | |  |  |
| Olfm1 |  | Noelin | |  |  |
| Olfm4 |  | Olfm4 protein | |  |  |
| Orm1 |  | Alpha-1-acid glycoprotein (AGP) (Fragment) | |  |  |
| Ovch2 |  | Isoform 2 of Ovochymase-2 | |  |  |
| P4hb |  | Protein disulfide-isomerase (Fragment) | |  |  |
| Pappa |  | Pappalysin-1 | |  |  |
| Parvb |  | Beta-parvin | |  |  |
| Patj |  | Isoform 4 of InaD-like protein | |  |  |
| Pcdh1 |  | Protocadherin 1 (Fragment) | |  |  |
| Pcsk9 |  | Proprotein convertase subtilisin/kexin type 9 | |  |  |
| Pcyox1 |  | Prenylcysteine oxidase 1 | |  |  |
| Pdcd6ip |  | Programmed cell death 6-interacting protein | | X |  |
| Pdha1 |  | Pyruvate dehydrogenase E1 component subunit alpha, somatic form, mitochondrial | |  |  |
| Pdhb |  | Pyruvate dehydrogenase E1 component subunit beta, mitochondrial | |  |  |
| Pdhx |  | Pyruvate dehydrogenase protein X component, mitochondrial | |  |  |
| Pdia3 |  | Protein disulfide-isomerase A3 | |  |  |
| Pecam1 |  | Isoform 4 of Platelet endothelial cell adhesion molecule | |  |  |
| Peg3 |  | Isoform 2 of Paternally-expressed gene 3 protein | |  |  |
| Pf4 |  | C-X-C motif chemokine | |  |  |
| Pfn1 |  | Profilin | | X |  |
| Pgk1 |  | Phosphoglycerate kinase 1 | | X |  |
| Pi16 |  | Isoform 2 of Peptidase inhibitor 16 | |  |  |
| Pigr |  | Polymeric immunoglobulin receptor | |  |  |
| Pip4k2a |  | Phosphatidylinositol 5-phosphate 4-kinase type-2 alpha (Fragment) | |  |  |
| Pkhd1l1 |  | Isoform 2 of Fibrocystin-L | |  |  |
| Pkm |  | Pyruvate kinase PKM | | X |  |
| Pla2g7 |  | Platelet-activating factor acetylhydrolase | |  |  |
| Pla2r1 |  | Isoform 2 of Secretory phospholipase A2 receptor | |  |  |
| Plek |  | Pleckstrin | |  |  |
| Plekho2 |  | Pleckstrin homology domain-containing family O member 2 | |  |  |
| Plg |  | Plasminogen | |  |  |
| Plin3 |  | Perilipin-3 | |  |  |
| Plin4 |  | Perilipin-4 | |  |  |
| Pltp |  | Phospholipid transfer protein | |  |  |
| Pm20d1 |  | N-fatty-acyl-amino acid synthase/hydrolase PM20D1 | |  |  |
| Pon1 |  | Serum paraoxonase/arylesterase 1 | |  |  |
| Postn |  | Periostin isoform M1 | |  |  |
| Ppia |  | Peptidyl-prolyl cis-trans isomerase A | | X |  |
| Ppic |  | Peptidyl-prolyl cis-trans isomerase C | |  |  |
| Ppp1ca |  | Serine/threonine-protein phosphatase PP1-alpha catalytic subunit | |  |  |
| Prdx1 |  | Peroxiredoxin-1 (Fragment) | | X |  |
| Prg4 |  | Proteoglycan 4 | |  |  |
| Pros1 |  | Vitamin K-dependent protein S | |  |  |
| Psap |  | Prosaposin (Fragment) | |  |  |
| Psma1 |  | Proteasome subunit alpha type | |  |  |
| Psma2 |  | Proteasome endopeptidase complex (Fragment) | |  |  |
| Psma3 |  | Proteasome endopeptidase complex | |  |  |
| Psma4 |  | Proteasome subunit alpha type (Fragment) | |  |  |
| Psma5 |  | Proteasome subunit alpha type | |  |  |
| Psma6 |  | Proteasome endopeptidase complex | |  |  |
| Psma7 |  | Proteasome endopeptidase complex | |  |  |
| Psmb1 |  | Proteasome subunit beta type-1 | |  |  |
| Psmb5 |  | Proteasome subunit beta type-5 | |  |  |
| Psmb8 |  | Proteasome subunit beta type | |  |  |
| Ptprb |  | Receptor-type tyrosine-protein phosphatase beta | |  |  |
| Ptprc |  | Receptor-type tyrosine-protein phosphatase C | |  |  |
| Ptprg |  | Receptor-type tyrosine-protein phosphatase gamma | |  |  |
| Ptprj |  | Receptor-type tyrosine-protein phosphatase eta | |  |  |
| Ptprz1 |  | Receptor-type tyrosine-protein phosphatase zeta, R-PTP-zeta, EC 3.1.3.48 (Fragment) | |  |  |
| Ptx3 |  | Pentraxin-related protein PTX3 | |  |  |
| Pzp |  | Pregnancy zone protein | |  |  |
| Q31152 |  | MHC class I Q4 beta-2-microglobulin (Qb-1) (Fragment) | |  |  |
| Q3U3I6 |  | Aspartyl aminopeptidase | |  |  |
| Q3UFL3 |  | Uncharacterized protein | |  |  |
| Q3UNR7 |  | Uncharacterized protein (Fragment) | |  |  |
| Q8BK55 |  | Uncharacterized protein (Fragment) | |  |  |
| Q8BMA4 |  | ARP2 actin-related protein 2 | |  |  |
| Q8CAU4 |  | Signaling lymphocytic activation molecule family member 1, isoform CRA_b | |  |  |
| Q8K1F1 |  | Anti-VIPase light chain variable region (Fragment) | |  |  |
| Q920E6 |  | Pterin-mimicking anti-idiotope kappa chain variable region (Fragment) | |  |  |
| Q920E7 |  | Pterin-mimicking anti-idiotope heavy chain variable region (Fragment) | |  |  |
| Q9D8L4 |  | Uncharacterized protein | |  |  |
| Q9JL80 |  | Anti-myosin immunoglobulin light chain variable region (Fragment) | |  |  |
| Qsox1 |  | Isoform 3 of Sulfhydryl oxidase 1 | |  |  |
| Rab11b |  | Ras-related protein Rab-11B (Fragment) | |  |  |
| Rab14 |  | RAB14 protein variant | |  |  |
| Rab1a |  | RAB1A, member RAS oncogene family | |  |  |
| Rab1b |  | Uncharacterized protein | |  |  |
| Rab27b |  | Ras-related protein Rab-27B | |  |  |
| Rab2a |  | Ras-related protein Rab-2A | |  |  |
| Rab7a |  | Ras-related protein Rab-7a | | X |  |
| Rac2 |  | Ras-related C3 botulinum toxin substrate 2 (Fragment) | |  |  |
| Rap1b |  | Ras-related protein Rap-1b | | X |  |
| Rasa3 |  | Uncharacterized protein | |  |  |
| Rbm6 |  | RNA-binding motif protein 6 | |  |  |
| Rdx |  | Radixin] | |  |  |
| Reln |  | Isoform 3 of Reelin | |  |  |
| Resf1 |  | Retroelement silencing factor 1 (Fragment) | |  |  |
| Rhog |  | Rho-related GTP-binding protein RhoG | |  |  |
| Rida |  | 2-iminobutanoate/2-iminopropanoate deaminase | |  |  |
| Rpl18 |  | 60S ribosomal protein L18 | |  |  |
| Rpl7 |  | 60S ribosomal protein L7 | |  |  |
| Rps13 |  | 40S ribosomal protein S13 | |  |  |
| Rras |  | Ras-related protein R-Ras (Fragment) | |  |  |
| Rsu1 |  | Ras suppressor protein 1 | |  |  |
| Rtn3 |  | Isoform 3 of Reticulon-3 | |  |  |
| S100a9 |  | Protein S100-A9 | |  |  |
| Saa4 |  | Serum amyloid A-4 protein | |  |  |
| Sardh |  | Sarcosine dehydrogenase, mitochondrial (Fragment) | |  |  |
| Scamp3 |  | Secretory carrier-associated membrane protein | |  |  |
| Scn3a |  | Sodium channel protein type 3 subunit alpha | |  |  |
| Sdcbp |  | Syntenin | | X |  |
| Sdha |  | Succinate dehydrogenase [ubiquinone] flavoprotein subunit, mitochondrial | |  |  |
| Sdhb |  | Succinate dehydrogenase [ubiquinone] iron-sulfur subunit, mitochondrial (Fragment) | |  |  |
| Sec16a |  | Protein transport protein Sec16A (Fragment) | |  |  |
| Selenbp1 |  | Methanethiol oxidase | |  |  |
| Selenop |  | Selenoprotein P (Fragment) | |  |  |
| Sell |  | L-selectin | |  |  |
| Serinc3 |  | Serine incorporator 3 | |  |  |
| Serpina10 |  | Isoform 2 of Protein Z-dependent protease inhibitor | |  |  |
| Serpina1b |  | Alpha-1-antitrypsin 1-2 | |  |  |
| Serpina1c |  | Alpha-1-antitrypsin 1-3 | |  |  |
| Serpina3k |  | Serine protease inhibitor A3K | |  |  |
| Serpina3n |  | Serine protease inhibitor A3N | |  |  |
| Serpina6 |  | Corticosteroid-binding globulin | |  |  |
| Serpinc1 |  | Antithrombin-III | |  |  |
| Serpind1 |  | Heparin cofactor 2 | |  |  |
| Serpinf2 |  | Alpha-2-antiplasmin | |  |  |
| Serping1 |  | Plasma protease C1 inhibitor | |  |  |
| Sftpd |  | Pulmonary surfactant-associated protein D | |  |  |
| Shroom3 |  | Protein Shroom3 | |  |  |
| Sis |  | Sucrase-isomaltase | |  |  |
| Skap2 |  | Isoform 2 of Src kinase-associated phosphoprotein 2 | |  |  |
| Slc1a5 |  | Amino acid transporter | |  |  |
| Slc25a3 |  | Phosphate carrier protein, mitochondrial | |  |  |
| Slc25a4 |  | ADP/ATP translocase 1 | |  |  |
| Slc2a3 |  | Solute carrier family 2, facilitated glucose transporter member 3 | |  |  |
| Slc3a2 |  | 4F2 cell-surface antigen heavy chain, 4F2hc (fragment) | | X |  |
| Slc44a2 |  | Choline transporter-like protein 2 | |  |  |
| Slc4a1 |  | Anion exchange protein | |  |  |
| Slc6a4 |  | Serotonin transporter (Fragment) | |  |  |
| Slc7a5 |  | Large neutral amino acids transporter small subunit 1 | |  |  |
| Slc9a3r1 |  | Na(+)/H(+) exchange regulatory cofactor NHE-RF1 | |  |  |
| Snap23 |  | Synaptosomal-associated protein (Fragment) | |  |  |
| Snrnp70 |  | U1 small nuclear ribonucleoprotein 70 kDa (Fragment) | |  |  |
| Sparcl1 |  | SPARC-like protein 1 | |  |  |
| Spg7 |  | Paraplegin | |  |  |
| Sptan1 |  | Isoform 2 of Spectrin alpha chain, non-erythrocytic 1 | |  |  |
| Srgn |  | Serglycin | |  |  |
| Srl |  | Isoform 2 of Sarcalumenin | |  |  |
| Steap3 |  | Metalloreductase STEAP3 (Fragment)] | |  |  |
| Stom |  | Erythrocyte band 7 integral membrane protein | |  |  |
| Stx12 |  | Syntaxin-12 | |  |  |
| Stxbp2 |  | Syntaxin-binding protein 2 | |  |  |
| Svep1 |  | Sushi, von Willebrand factor type A, EGF and pentraxin domain-containing protein 1 | |  |  |
| Tagln2 |  | Transgelin-2 | |  |  |
| Tcn2 |  | Transcobalamin-2 | |  |  |
| Tf |  | Serotransferrin | |  |  |
| Tfg |  | Trk-fused gene (Fragment) | |  |  |
| Tfrc |  | Transferrin receptor protein 1] | |  |  |
| Tgfb1 |  | Transforming growth factor beta-1 | |  |  |
| Tgfbi |  | Transforming growth factor-beta-induced protein ig-h3, Beta ig-h3 | |  |  |
| Thbs1 |  | Thrombospondin-1 | |  |  |
| Thbs4 |  | Thrombospondin-4 | |  |  |
| Thy1 |  | Thy-1 membrane glycoprotein (Fragment) | |  |  |
| Tln1 |  | Talin-1 OS=Mus musculus | | X |  |
| Tmem131l |  | Isoform 3 of Transmembrane protein 131-like | |  |  |
| Tnc |  | Isoform 2 of Tenascin | |  |  |
| Tnc |  | Tenascin | |  |  |
| Tns4 |  | Tensin-4 | |  |  |
| Tpm3 |  | Tropomyosin alpha-3 chain | |  |  |
| Tpm4 |  | Tropomyosin alpha-4 chain | |  |  |
| Trip12 |  | E3 ubiquitin-protein ligase TRIP12 (Fragment) | |  |  |
| Trpm3 |  | Transient Receptor Potential Cation Channel Subfamily M Member 3 | |  |  |
| Try10 |  | MCG140784 | |  |  |
| trypsinogen |  | Trypsinogen 5 | |  |  |
| Tspan9 |  | Tetraspanin (Fragment) | |  |  |
| Ttr |  | Transthyretin | |  |  |
| Tuba1c |  | Tubulin alpha-1C chain | |  |  |
| Tuba4a |  | Tubulin alpha-4A chain | |  |  |
| Tubb1 |  | Tubulin beta-1 chain | |  |  |
| Tubb5 |  | Tubulin beta-5 chain | |  |  |
| Uba1 |  | Ubiquitin-like modifier-activating enzyme 1 | | X |  |
| Ubc |  | Ubiqutin subunit 1 (Fragment) | |  |  |
| Upf2 |  | UPF2 regulator of nonsense transcripts homolog (Yeast) | |  |  |
| Uqcrb |  | Cytochrome b-c1 complex subunit 7 | |  |  |
| Uqcrc1 |  | Cytochrome b-c1 complex subunit 1, mitochondrial | |  |  |
| Uqcrc2 |  | Cytochrome b-c1 complex subunit 2, mitochondrial | |  |  |
| V165-D-J-C mu |  | V165-D-J-C mu protein (Fragment) | |  |  |
| Vamp3 |  | Vesicle-associated membrane protein 3 | |  |  |
| Vcam1 |  | Vascular cell adhesion protein 1 | |  |  |
| Vcan |  | Versican | |  |  |
| Vcl |  | Vinculin | |  |  |
| Vcp |  | Transitional endoplasmic reticulum ATPase, TER ATPase, EC 3.6.4.6 | |  |  |
| Vdac1 |  | Voltage-dependent anion-selective channel protein 1 | |  |  |
| Vtn |  | Vitronectin | |  |  |
| Vwf |  | von Willebrand factor | |  |  |
| Wasf2 |  | Uncharacterized protein (Fragment) | |  |  |
| Wdr1 |  | WD repeat-containing protein 1 | |  |  |
| X5J4E9 |  | IgM heavy chain VDJ region (Fragment) | |  |  |
| X5J4K1 |  | IgA heavy chain VDJ region (Fragment) | |  |  |
| X5J4K5 |  | IgA heavy chain VDJ region (Fragment) | |  |  |
| X5J4Q4 |  | IgM heavy chain VDJ region (Fragment) | |  |  |
| X5J4V9 |  | IgA heavy chain VDJ region (Fragment) | |  |  |
| X5J4Y7 |  | IgM heavy chain VDJ region (Fragment) | |  |  |
| X5J524 |  | IgM heavy chain VDJ region (Fragment) | |  |  |
| X5J533 |  | IgG1 heavy chain VDJ region (Fragment) | |  |  |
| X5J574 |  | IgM heavy chain VDJ region (Fragment) | |  |  |
| X5J577 |  | IgM heavy chain VDJ region (Fragment) | |  |  |
| X5J5B7 |  | IgM heavy chain VDJ region (Fragment) | |  |  |
| X5J5C1 |  | IgM heavy chain VDJ region (Fragment) | |  |  |
| X5J5C8 |  | IgM heavy chain VDJ region (Fragment) | |  |  |
| X5J5E8 |  | IgG1 heavy chain VDJ region (Fragment) | |  |  |
| X5J5H4 |  | IgA heavy chain VDJ region (Fragment) | |  |  |
| X5J5K8 |  | IgM heavy chain VDJ region (Fragment) | |  |  |
| X5J5N4 |  | IgM heavy chain VDJ region (Fragment) | |  |  |
| Xylt2 |  | Protein xylosyltransferase | |  |  |
| Ywhab |  | Isoform Short of 14-3-3 protein beta/alpha | |  |  |
| Ywhah |  | 14-3-3 protein eta | |  |  |
| Ywhaz |  | 14-3-3 protein zeta/delta (Protein kinase C inhibitor protein 1, KCIP-1) (Fragment) | | X |  |
|  |  |  | |  |  |
